# Multiple MAPK Cascades Regulate the Transcription of *IME1*, the Master Transcriptional Activator of Meiosis in *Saccharomyces cerevisiae*

**DOI:** 10.1371/journal.pone.0078920

**Published:** 2013-11-13

**Authors:** Smadar Kahana-Edwin, Michal Stark, Yona Kassir

**Affiliations:** Department of Biology, Technion - Israel Institute of Technology, Haifa, Israel; Tulane University Health Sciences Center, United States of America

## Abstract

The choice between alternative developmental pathways is primarily controlled at the level of transcription. Induction of meiosis in budding yeasts in response to nutrient levels provides a system to investigate the molecular basis of cellular decision-making. In *Saccharomyces cerevisiae*, entry into meiosis depends on multiple signals converging upon *IME1*, the master transcriptional activator of meiosis. Here we studied the regulation of the *cis*-acting regulatory element Upstream Activation Signal (UAS)ru, which resides within the *IME1* promoter. Guided by our previous data acquired using a powerful high-throughput screening system, here we provide evidence that UASru is regulated by multiple stimuli that trigger distinct signal transduction pathways as follows: (*i*) The glucose signal inhibited UASru activity through the cyclic AMP (cAMP/protein kinase A (PKA) pathway, targeting the transcription factors (TFs), Com2 and Sko1; (*ii*) high osmolarity activated UASru through the Hog1/mitogen-activated protein kinase (MAPK) pathway and its corresponding TF Sko1; (*iii*) elevated temperature increased the activity of UASru through the cell wall integrity pathway and the TFs Swi4/Mpk1 and Swi4/Mlp1; (*iv*) the nitrogen source repressed UASru activity through Sum1; and (*v)* the absence of a nitrogen source was detected and transmitted to UASru by the Kss1 and Fus3 MAPK pathways through their respective downstream TFs, Ste12/Tec1 and Ste12/Ste12 as well as by their regulators Dig1/2. These signaling events were specific to UASru; they did not affect the mating and filamentation response elements that are regulated by MAPK pathways. The complex regulation of UASru through all the known vegetative MAPK pathways is unique to *S. cerevisiae* and is specific for *IME1*, likely because it is the master regulator of gametogenesis.

## Introduction

Transcriptional regulation is the key mechanism that controls cell fate in response to internal and external stimuli. This is often achieved through a transcriptional regulatory cascade controlled by a master regulator [1], [2]. The master regulator is essential for cells to initiate a specific developmental pathway at the correct time and place. The identification of the signal transduction pathways and their components that regulate gene transcription (expression) is therefore essential for understanding how cell fate is determined.

The budding yeast *Saccharomyces cerevisiae* provides an experimental system for studying developmental decisions made by eukaryotic cells in response to nutrient deprivation. In the presence of any carbon and nitrogen sources cells vegetative growth is in oval shape. This organism prefers glucose as a carbon source and has evolved diverse regulatory mechanisms to ensure its survival in nature where the levels of glucose fluctuate widely. In response to nitrogen depletion, diploid *S. cerevisiae* has two alternative developmental pathways. The absence of glucose triggers cells to undergo meiosis and spore formation, which is referred to as sporulation (reviewed in [3], [4]), whereas the presence of glucose leads to growth as pseudohyphae (reviewed in [5]). Both of these developmental decisions, which are further influenced by mating type, are mainly controlled by the cyclic AMP (cAMP)/protein kinase A (PKA) pathway (reviewed in [6], [7]).

*IME1*, the master regulator of meiosis in budding yeast ( [8], reviewed in [3], [9]) is regulated by distinct signal transduction pathways that primarily control its transcription ([4], [6], [8], [10]–[12]). *IME1* transcription is repressed when glucose is provided as the sole carbon source; conversely, transcription is induced in the presence of acetate and no other carbon source [8]. Nitrogen depletion leads to a transient induction of *IME1* transcription, but only in cells expressing both the *MAT* **a** and *MAT*α alleles [8].

*RME1* is a transcription factor that inhibits *IME1* transcription in haploid cells of the *MAT* **a** or *MATα* mating types ( [13], [14]; reviewed in [3], [9]) but is not expressed by *MAT* **a/** *MATα* diploids [15], [16]. *RME1* positively regulates the expression of a long noncoding (lnc) RNA in cells expressing the haploid *MAT* **a** or *MAT*α mating types but not in diploid *MAT* **a**/α cells [17]. The lncRNA (*IRT1*) inhibits *IME1* expression in *cis* at the *IME1* promoter by inducing the formation of a repressive chromatin structure comprising Set2 histone methyltransferase and the Set3 histone decarboxylase [17].

The 2.1 kb *IME1* promoter is unusually long compared with other *Saccharomyces* upstream regulatory elements (approximately 437 bp; *Saccharomyces* Genome Database, http://www.yeastgenome.org/) and comprises at least 10 distinct positive- and negative-control elements (reviewed in [9]). We previously focused on dissecting each control element and were able to identify parallel pathways that regulate the carbon source-responsive elements within *IME1* promoter [9], [18]. We analyzed in detail one element, IREu whose function is regulated by glucose through the cAMP/PKA pathway [11], [18]–[20]. In the present study, we extend our detailed analysis of the *IME1* promoter by molecularly dissecting the upstream activation sequence (UASru), a glucose-responsive regulatory element that is essential for the robust transcription in the absence of glucose [9]. Guided by our previous data generated using a high-throughput functional screen (Reporter-Synthetic Genetic Array, R-SGA) [20], our present analysis reveals that the activity of the UASru is regulated by high osmolarity, temperature, and nitrogen through each of the four distinct mitogen-activated protein kinase (MAPK) pathways present in budding yeasts (reviewed in [5]). We demonstrate further that the signal emitted by the carbon source also traverses the cAMP-PKA pathway to engage two distinct transcription factors.

## Materials and Methods

### Strains and Plasmids

Table S1 lists the plasmids used in this study. Table S2 lists the relevant genotype of the strains. The genotypes of the strains and copy number of inserted genes were verified using polymerase chain reaction (PCR) and quantitative PCR analyses, respectively. A detailed description of plasmid and strain constructions is available upon request.

### Media and Growth Conditions

SD, minimal glucose, SLAD (synthetic glucose medium with glutamic acid as a nitrogen source), PSP2 (SA, minimal acetate), SPM, and SPO (nitrogen and glucose starvation media to induce meiosis and sporulation in liquid and plates, respectively) media were prepared as reported [21]–[23]. Meiosis was induced by growing cells in PSP2 to early exponential stage (0.8–1.2×10^7^ cells/ml), harvesting, washing once with water, and re-suspending in SPM. The β-galactosidase activity was assayed as described [24]. Unless otherwise indicated in the Figure Legends, cells were cultured at 30°C; 1×10^7^ or 3×10^8^ cells were harvested during exponential phase for β-galactosidase or ChIP assays, respectively.

### Quantitative Analysis of RNA Levels

RNA was extracted from 10^8^ cells using the hot acidic phenol method [25]. Approximately 1 µg of total RNA was used for reverse transcription reactions with random hexamer primers and Invitrogen SuperScript® Reverse-T Transcriptase III (Life Technologies). The cDNA products were used as templates for real-time (RT)-PCR analysis (nPCR) according to the manufacturer’s instructions (ABGene, Surrey, U.K.).

Primers: UASru: 5′CGTTGATGTCATCCGCTATT-3′and either 5′- GACCCAAGAAGCCACCATGA-3′ for the genomic sequence, or 5′-CATACCTCGACATCACATGCT-3′ for *UASru-his4-lacZ*; *ACT1*∶**5′-**ATCACCGCTTTGGCTCCAT-3′ and 5′-CCAATCCAGACGGAGTACTTTCTT-3′.

### Chromatin Immunoprecipitation (ChIP)

ChIP assays were performed essentially as described [10]. Thirteen or six Myc-tag sequences were ligated to *COM2, SWI4, TEC1, DIG1,* and *STE12* or to *SKO1, HOG1, SUM1, MLP1, MPK1*, respectively, and were inserted in place of their cognate endogenous genomic allele, and their expression was regulated by their natural promoters. Following IP, 100 ng genomic DNA was analyzed using qPCR for specific (IP), nonspecific (whole-cell lysate, WCE), and IP without primary antibody (IP w/o Ab) according to the manufacturer’s instructions (ABGene, Surrey, U.K.). Three primer sets were used for qPCR as follows: 1) UASru (*UASru-his4-lacZ* chimera). This construct, integrated into the genome, was used to examine the binding of the various TFs. This construt was also used to examine its expression level in response to various perturbations. This allowed us to conclude about binding to the specific tested element, rather than adjacent regions within *IME1* promoter; 2) control *TEL1* or *POL1*; and 3) positive control (a gene known to be directly regulated by the examined TF, as described in the Results). The extent of enrichment was calculated as the ratio of specific (IP) to nonspecific (WCE) DNA, and the data were normalized to that of IP w/o Ab. Antibodies: Mouse anti Myc epitope (9E11) (Santa Cruz), 0.33 µg per sample; mouse anti-HA epitope (12CA5) (Roche), 0.8 µg per sample. Primers: Table S3 describes the primers used in this study for ChIP assay.

## Results

### The Functions of UASru

**Figure 1:**
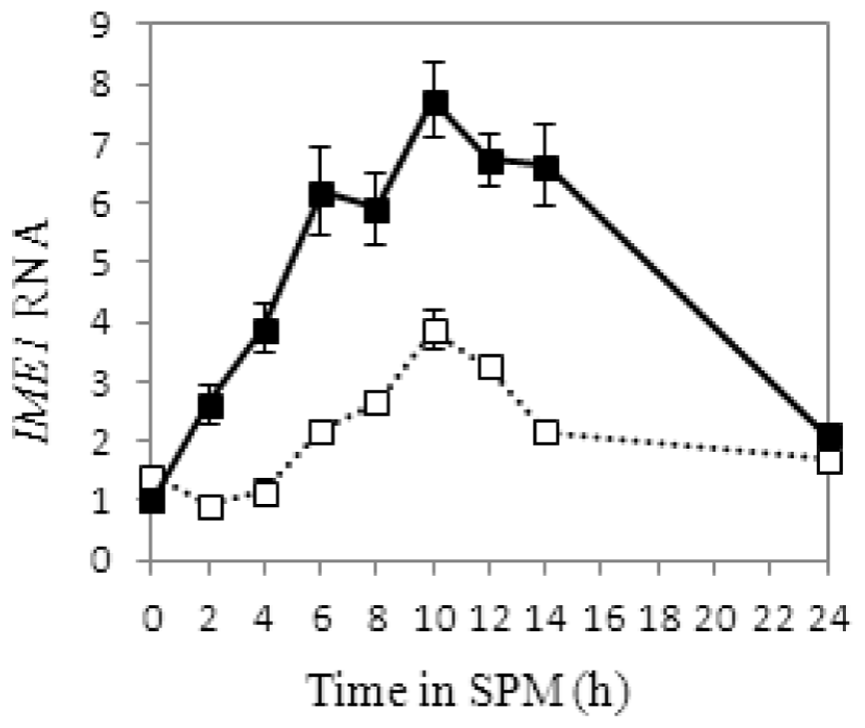
UASru functions throughout meiosis as a positive element in the *IME1* promoter. The isogenic diploid strains *ime1*Δ*/IME1* (Y1639, filled squares) and *ime1*Δ*UASru*/*ime1*Δ (Y1741, open squares dashed line). The *IME1* RNA levels shown are relative to that of *ACT1*, normalized to the wt value at time 0. The results represent the average of at least three independent transformants.

UASru is located between nucleotide positions −1198 to −1370 from the initiation codon of the *IME1* open reading frame [9]. To validate its enhancer function in the context of the *IME1* promoter, we deleted UASru from the genome. This decreased the levels of *IME1* mRNA by approximately 2-fold throughout the meiotic pathway (Fig. 1). This effect, although small, is significant, because the transcription of *IME1* is regulated by multiple UASs [9].

To determine whether this effect was caused by inhibition of transcription, increased mRNA turnover, or both, we deleted UASru from a chimeric *ime1-lacZ* reporter in which *lacZ* transcription is driven by the *IME1* promoter. Cells deleted for UASru and grown in SA (acetate without glucose) and SPM (acetate without glucose or a nitrogen source) (Table 1) expressed lower levels of β-galactosidase in comparison to wild-type. In contrast, no effect was detected (Table 1) in SD, likely because glucose represses *IME1* transcription is mediated by at least two additional distinct elements [9]. These data support the conclusion that UASru functions as a positive element when glucose and a nitrogen source are absent. Moreover, the increase in reporter expression in cells grown SPM compared with that in SA suggests that the nitrogen depletion signal is transmitted to *IME1* through an additional element (not UASru), consistent with the results of our previous study [18].

**Table 1:** UASru is required for the transcription of *IME1* in the absence of glucose.

Reporter gene	Medium			
	SD	SA	SPM 3 hrs.	SPM 6 hrs.
	β-galactosidase activity (Miller units)			
*ime1*-*lacZ*	0.18±0.01	0.51±0.05	70.41±2.92	135.69±1.92
ime1ΔUASru-la*cZ*	0.17±0.01	0.30±0.03	38.65±9.04	97.98±8.51
*UASru-his4-lacZ*	29.00±1.10	149.07±5.90	NT	117.77±3.78
*his4-lacZ*	1.00±0.50	0.90±0.20	NT	1.50±0.60

The results represent the average of the data for three independent transformants ± the standard deviation. Strains used were as follows: Y1623, Y1624, Y1685, and Y422-R are wt diploids carrying *IME1*-*lacZ*, *IME1*ΔUASru-*lacZ*, *UASru-his4-lacZ*, or *his4-lacZ,* respectively. NT – Not Tested.

**Figure 2:**
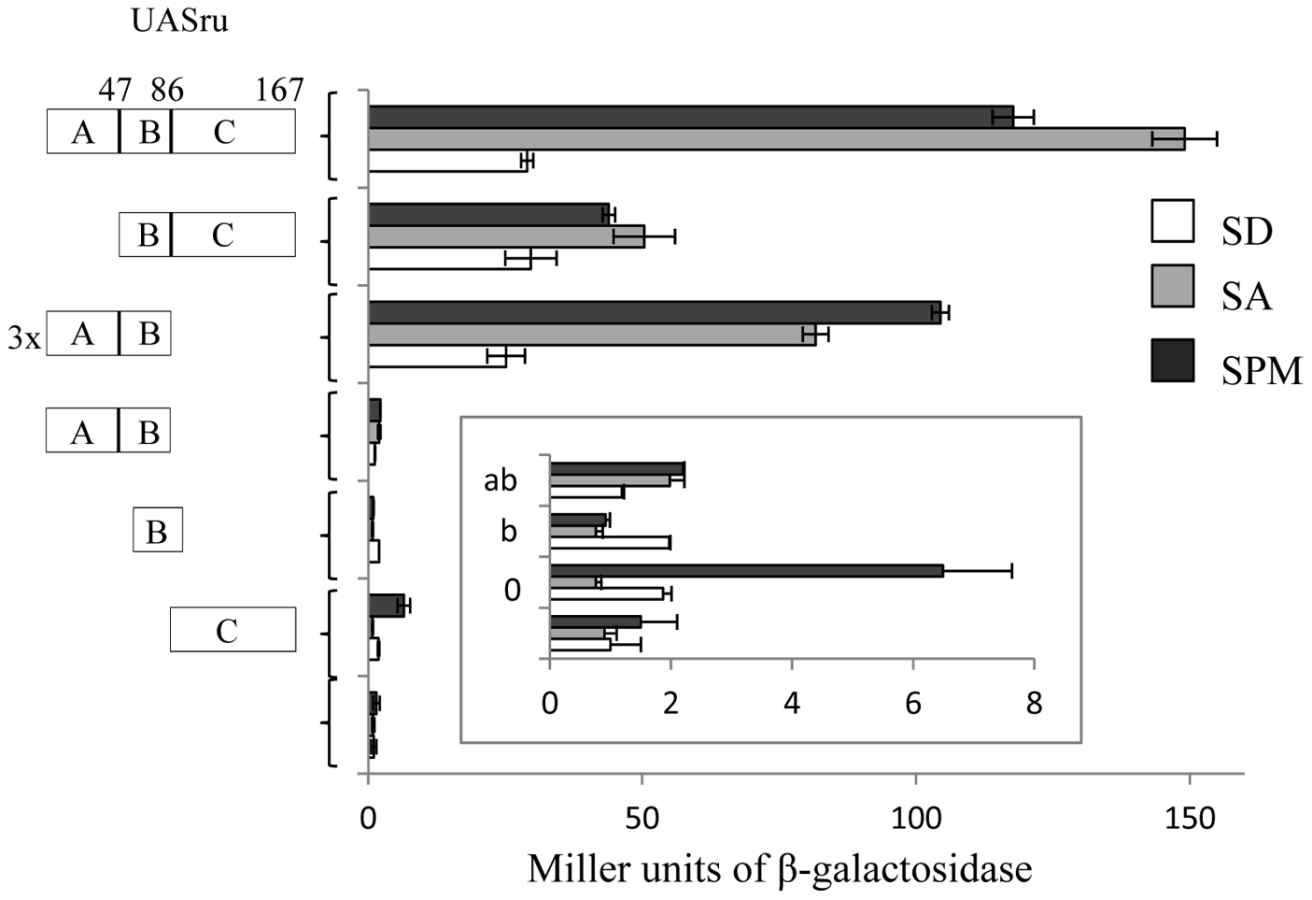
UASru comprises at least two distinct UAS elements that are controlled by carbon and nitrogen sources. A series of *his4-lacZ* reporters carrying different segments of UASru were integrated within genomic *LEU2*. Proteins were extracted from cells (1×10^7^ cells/ml) cultured in SD, SA or 6 hours in SPM. The results represent the average of at least three independent transformants. The isogenic diploid strains used were as follows: Y1214 (*UASru-lacZ*), Y1625 (*UASru-BC-lacZ*), Y1697 (*UASru-C-lacZ*), Y1686 (3x [*UASru-AB*]*-lacZ*), Y1670 (*UASru-AB-lacZ),* Y1669 (*UASru-B-lacZ*), and Y422-R (*his4-lacZ).*

Because the UASru element is relatively large (173 base pairs (bp), it may contain multiple UAS, or URS (Upstream Repression Sequence) elements, or both, that are mutually inhibitory. To ensure that only carbon sources regulate UASru, we divided it into three segments, designated A, B, and C (see Fig. 2). We generated constructs containing different or combined segments inserted upstream of an inactive *his4-lacZ* reporter (this reporter includes only a TATA box, without any UAS). The rational for using heterologous reporters rather than observing an *IME1* expression levels, was to overcome the presence of the additional elements in its promoter. These elements could mask our ability to observe response to specific signals. The activity of the ABC construct (the entire UASru) was as reported [9], namely low in SD and increased activity in SA and SPM (Fig. 2). The activity of the AB construct was similar, higher in cells grown in SA and SPM compared with that in SD. In contrast, the activity of the B region construct was lower in cells grown in SA compared with that in SD (Fig. 2). These data suggest that the A region supports the activity of the UASru-AB to a greater extent than B.

The UAS activity of the C construct was relatively low compared with those of the others. Moreover, its activity was increased 6.5-times when cells were grown in SPM (Fig. 2), suggesting that the absence of a nitrogen source activates this element or that it functions as a URS in the presence of nitrogen. The latter possibility is supported by findings that insertion of UASru-C upstream of a *HIS4* UAS in the *his4-lacZ* reporter (strain Y2029 compared with Y2028) resulted in 10.7, 11.2, and 22-fold reductions in reporter expression in SD, SA, and SPM media, respectively (data not shown). These results support the conclusion that UASru-C acts as a negative regulatory element. Further work is required to reveal why nitrogen depletion did not promote the expression of this reporter.

The activity of UASru-BC was similar to that of UASru-ABC and UASru-AB. Because this construct was not significantly affected by the presence of a nitrogen source, we suggest that a sequence in region B, or one that was disrupted or removed from the B and C elements, masked detection of a response of the BC element to a nitrogen source.

### Identification of Pathways that Transmit Glucose and Nitrogen-source Signals to the UASru

We previously used the R-SGA approach [20] to screen the viable deletion array of ∼4500 genes for mutants that affect the transcription of GFP reporter gene, whose expression was separately controlled by several distinct UAS and URS elements from *IME1* promoter. We normalized the expression of these reporters to a constitutively expressed RFP reporter. The data was transformed to Z-scores, and P-values on the basis of a normal distribution (for details see [20], [26]). A cutoff of 10% was used to identify putative regulators. In our previous study we focused on the identification of transcription factors, and reported four genes (*TEC1*, *SUM1*, *SWI4*, and *COM2*) that affect the activity of UASru [20]. In the present study, we used the R-SGA data to identify all pathways that act through the UASru. The following had a significant effect: cAMP/PKA, osmotic stress, and CWI (Cell Wall Integrity). We also identified TFs likely targeted by these pathways based on bioinformatic or genome-wide association analysis [20] (Table 2). Pathway analysis is presented in the following sections.

**Table 2:** The role of signal transduction pathways and putative TFS on the activity of UASru.

	Signaling genes			TF Genes		
Pathway	Gene	Rate	P-value	Gene	Rate	P-value
PKA	*GPR1*	2.20	5.19E-023	*COM2*	2.24	4.39E-068
	*RAS1*	1.44	−1.27E-008	*SKO1*	1.24	−0.0322
	*RGS2*	1.41	4.62E-007	*MSN2*	1.22	−0.0394
	*TPK1*	1.56	1.24E-010	*MSN4*	1.13	−0.1232
	*TPK2*	1.26	−5.46E-007	*SOK2*	1.04	0.1294
	*ASC1*	1.35	−0.004235	*FLO8*	1.03	0.000107
	*PDE1*	1.91	4.24E-016	*SFL1*	1.02	−0.0266
	*RIM11*	1.52	−1.98E-010			
	*RPI1*	0.75	−4.05E-006			
Osmotic stress	*HOG1*	1.43	3.88E-006	*SKO1*	1.24	−0.0322
	*OPY2*	0.79	−0.00499	*MSN1*	0.78	0.000235
	*PTC3*	0.66	−1.05E-010	*MSN2*	1.22	−0.0394
	*SSK1*	1.55	0.00063	*MSN4*	1.13	−0.1232
	*SSK22*	1.55	2.20E-008	*HOT1*	1.04	0.2254
				*SMP1*	0.98	−0.177
CWI	*PKH1*	1.74	1.02E-015	*SWI4*	0.58	−3.32E-028
	*PKH3*	0.75	−5.96E-006	*SWI6*	NT	2.61E-006
	*TUS1*	1.89	1.27E-015	*RLM1*	1.06	−0.1929
	*WSC2*	1.78	1.13E-016			
	*MTL1*	1.95	5.88E-017			
	*RPI1*	0.74	−4.05E-006			
	*MLP1*	1.17	0.0043			

Rate (log2 of gene expression vs. background control expression level) was determined using the R-SGA assay [20]. P-values were calculated on the basis of a normal distribution. NT – Not tested.

### The cAMP/PKA Pathway

The R-SGA screen identified nine genes that encode components of the cAMP/PKA pathway (Table 2) [27]–[29]. We assessed the involvement of this pathway in regulating the activity of UASru by examining the effects of *GPR1* (G protein-coupled receptor), *RAS2* (small GTP-binding protein that activates adenylate cyclase), and *CDC25* (Ras2 guanine nucleotide exchange factor) on the expression of a *UASru-lacZ* reporter construct. Deletion of *GPR1* or the *SH3* domain of *CDC25* (required for its association with adenylate cyclase [30] resulted in an increase in reporter expression in SD and SA media (Table 3). These results indicate that this pathway inhibits the activity of UASru in the presence of glucose and acetate. Deletion of *RAS2* had a similar effect but only in SD (Table 3) for unknown reasons.

**Table 3:** The role of the cAMP/PKA pathway and Com2 in regulating the activity of UASru.

Genotype	SD	SD Stat	SA	SPM 6 h
	β-galactosidase activity (Miller units)			
wt diploid, *UASru-lacZ*	9.50±0.72	NT	96.2±3.10	94.3±4.44
*gpr1*Δ*/gpr1*Δ, *UASru-lacZ*	20.50±1.30	NT	227.6±8.53	225.7±6.12
*ras2*Δ*/ras2*Δ, *UASru-lacZ*	20.90±0.94	NT	86.9±3.65	92.5±0.58
*cdc25*Δ*SH3, UASru-lacZ*	18.60±0.95	NT	247.8±0.85	NT
wt haploid, 3x *[UASru-AB]-lacZ*	35.69±5.35	48.40±14.72	134.20±0.18	127.55±9.05
*com2*Δ, *3x [UASru-AB]-lacZ*	51.93±6.93	196.18±21.71	NT	NT
*com2S164AS88A, 3x [UASru-AB]-lacZ*	50.02±2.35	70.98±0.74	NT	NT
wt haploid, *UASru-lacZ*	35.30±3.0	NT	133.8±10.0	NT
*sko1*Δ, *UASru-lacZ*	83.50±8.4	NT	143.3±11.2	NT

*Com2*Δ cells did not grow on acetate medium. Therefore, *com2* mutants and their isogenic wt strain were grown in SD medium to either logarithmic stage (SD) or stationary stage (SA). The results are expressed as relative β-galactosidase activity and represent the average data for three independent transformants. Strains used were as follows: wt (Y1265) and its isogenic strain *gpr1*Δ*/gpr1*Δ (Y1267), *ras2*Δ*/ras2*Δ (Y1270), *cdc25*Δ/*cdc25*Δ carrying *CDC25ΔSH3* on a *URA3* CEN vector (Y1338-2), a wt haploid (Y1890) and its isogenic strains *com2*Δ (Y1968) and *com2S164AS88A* (Y2052), a wt haploid (Y1214) strain and its isogenic strain sko1Δ (Y2063). These strains carry the *lacZ* reporter genes integrated within genomic *LEU2*. NT – Not Tested.

Signaling through the cAMP/PKA pathway targets the TFs Msn2, Msn4, Sok2, Sfl1, and Flo8 [11], [19], [31]–[34]. However, individual deletion of their respective genes did not affect the expression of *UASru-GFP* (Table 2). The results of R-SGA analysis indicate that Sko1 and Com2 may be engaged by signaling through the cAMP/PKA pathway. The findings supporting this conclusion are as follows: (*i*) The expression of *UASru-GFP* was significantly increased in *COM2* deletion mutant to the level expressed by *gpr1*Δ mutants (Table 2); A slight increase was also observed for *SKO1* deletion (Table 2) (*ii*) the A region of UASru, which is regulated by glucose (Fig. 2), has an imperfect putative Sko1-binding site as well as two imperfect putative Com2-binding sites; (*iii*) Sko1 is a target of the cAMP/PKA pathway, and its transcriptional repressor activity is increased upon phosphorylation by PKA [35]; and (*iv*) data acquired using global mass spectrometry detected Com2-phosphorylated peptides with sequences matching the canonical PKA phosphorylation site [36].

**Figure 3:**
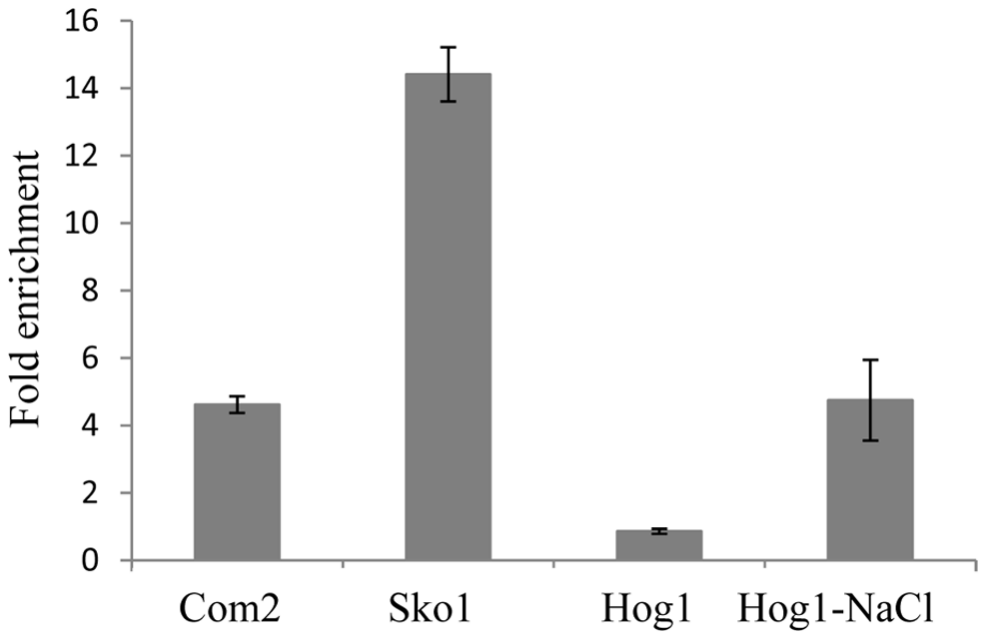
Com2, Sko1, and Hog1 bind UASru. Samples for ChIP assays were taken from 3×10^8^ logarithmic cells grown in SD. For the Com2-tagged strain, a sample was taken from cells grown in SA. In addition, for the Hog1-tagged strain, samples were taken before and after incubation in 0.4 M NaCl for 10 min. Endogenous *UASru* and *POL1* sequences were amplified using qPCR. Binding to UASru was calculated as the ratio of UASru to *POL1* DNAs and normalized to the values for IP with no antibody. The results represent the average of data for three independent colonies. Strains used were as follows: Y2032, Y2037, and Y2045, carrying *HOG1-6HA, COM2-13myc*, and *SKO1-6HA*, respectively.

Deletion of *SKO1* did not significantly affect the activity of UASru in the R-SGA assay (Table 2). Nonetheless, because of the presence of an imperfect Sko1-binding site in UASru (TGAtGTCA versus TGACGTCA) [37], and because it is targeted by Hog1 MAPK (see below), we directly examined its role in regulating UASru. Deleting *SKO1* induced a 2-fold increase in the expression of *UASru-lacZ* when cells were cultured in SD but not in SA (Table 3). This result is consistent with findings that PKA activity increases in the presence of glucose (reviewed in [38]). A significant enrichment of Sko1 binding to UASru was detected using qChIP in cells grown in SD (Fig. 3).

*COM2* deletion mutants failed to grow on acetate as the sole carbon source (data not shown). To assess whether Com2 regulates UASru, we tested cells grown to logarithmic or stationary phase in SD (stationary phase cells were designated SD-Stat, a stage in which glucose was depleted by growth). Deletion of *COM2* induced an increase in *UASru-AB-lacZ* expression in both cultures (Table 3), suggesting that Com2 repressed transcription. To determine whether PKA modulates the activity of Com2, we constructed a *com2* allele in which the two putative PKA serine phosphorylation sites S88 and S164 were mutated to alanine. Cells expressing this mutant protein expressed elevated levels of the reporter gene (Table 3). Although indirect, these data are consistent with the conclusion that PKA phosphorylates Com2. Reporter expression by the *COM2* deletion mutant compared with the alanine mutant was similar in SD but not in SD-Stat (Table 3), suggesting that Com2 binds the UASru under all growth conditions, and that in its absence, a positive regulator binds more effectively and activates transcription. Consistent with this hypothesis are findings that in qChIP assays, Com2 specifically bound UASru in cells grown in SA (Fig. 3).

### The Osmotic Stress Pathway

The R-SGA screen identified five genes that encode components of the osmotic stress pathway (Table 2) [20], including Sko1, which is regulated by this pathway [5]. We hypothesized, therefore, that Sko1 may also transmit an osmotic stress signal to UASru (not only a glucose signal). We examined if the activity of UASru responds to osmotic stress. Adding 1M NaCl to cultures of wild-type (wt) cells increased the activity of UASru (Table 4). In contrast, deletion of *HOG1* (*hog1Δ/hog1Δ*) reduced UASru activity, particularly in cells cultured in SD (Table 4). NaCl kills this mutant strain because it lacks Hog1, and therefore was not tested. Surprisingly, opposite results were obtained when the effect of Hog1 was examined in the R-SGA and the lacZ assays: deletion of *HOG1* increased the expression of *UASru-GFP* (Table 2) but decreased expression of *UASru-lacZ* (Table 4). The reason for this discrepancy is unknown. qChIP assays demonstrated specific binding of Hog1 to UASru after exposure to NaCl (Fig. 3; [39]; and F. Possas, personal communication). On the other hand, Sko1 binding occurred in the absence of NaCl (Fig. 3), suggesting that Hog1 does not recruit Sko1 to UASru, as previously reported [5], and that Hog1 is not required for the binding and repression activity of Sko1. This is consistent with findings that phosphorylation of Sko1 by Hog1 inhibits repression of gene expression by Sko1 [40].

**Table 4:** Osmotic stress and Hog1 induce the activity of UASru.

Genotype	Treatment	SD	SA
		β-galactosidase activity (Miller units)	
HOG1/HOG1	None	30.51±1.03	119.73±3.69
HOG1/HOG1	1M NaCl	55.34±6.80	219.19±4.05
*hog1*Δ*/hog1*Δ	None	15.45±0.21	90.07±5.83
*kss1*Δ/*kss1*Δ	None	34.72±3.18	NT
*kss1*Δ/*kss1*Δ	1M NaCl	57.06±3.65	NT
*HOG1*	None	35.3±3.0	133.8±10.0
*HOG1*	0.4 M NaCl	78.8±5.9	NT

Cells were cultured in either SD or SA with or without 1M NaCl, or following exposure to 0.4 M NaCl and assayed for β-galactosidase activity. The results represent the average of data for three independent transformants. Strains used were diploid wt (Y1721) and its isogenic *hog1*Δ*/hog1*Δ (Y1908) and *kss1*Δ/*kss1*Δ (Y1909) strains, and a haploid wt (Y1214). All strains carried UASru-*lacZ* integrated within genomic *LEU2*. NT – Not Tested.

The effect of Hog1 on UASru activity could also be indirect and mediated by Kss1, which is inappropriately activated by the addition of NaCl [41]. However, the osmotic response of UASru was also evident in a *KSS1* deletion mutant (Table 4)., suggesting, as described above that the effect of Hog1 is direct as it binds UASru. Hog1 also affects the activity of the transcriptional activators Hot1, Msn1, Smp1, Msn2, and Msn4 [5]. Deletion of these genes did not significantly affect the expression of *UASru-GFP* (Table 2). In addition, potential binding sites within UASru are not present. Therefore, further experiments were not conducted.

### The CWI Pathway

The R-SGA screen identified six genes that are components of the CWI pathway (Table 2) [5]. Because the transcription of *IME1* is regulated by G1 arrest and elevated temperatures [42], the CWI pathway might transmit either of these signals to UASru. Treatment of *MAT* **a** haploid cells with α-factor resulted in G1 arrest; however, the level of expression of *UASru-lacZ* did not increase, but the expected increase in the expression of *fus1-lacZ* was detected (Table 5). In contrast, a shift to 37°C induced a significant increase in the expression of *UASru(AB)-lacZ* (Table 6), suggesting that the CWI pathway transmits a temperature signal that affects the activity of UASru.

**Table 5:** The α-factor does not regulate the activity of UASru.

	*UASru-lacZ*		*FUS1-lacZ*	
Medium	− α-factor	+ α-factor	− α-factor	+ α-factor
	β-galactosidase activity (Miller units)			
SD	107.41±9.13	98.88±2.01	0.70±0.09	110.78±4.35
SA	275.70±1.87	269.43±19.55	0.62±0.04	14.86±0.07
SPM 6 h	186.24±1.38	199.95±2.33	1.33±0.04	2.65±0.09

The α-factor was added to a final concentration of 5 µg/ml for 2 h. The β-galactosidase activity represents the average data from three independent transformants. Strains used were as follows: haploid *MAT*a (Y1064) carrying either UASru-*lacZ* integrated within genomic *LEU2* (Y1214) or *FUS1-lacZ* cloned into a CEN vector (YCp1174).

**Table 6:** The activity of UASru is regulated by temperature.

Time (h)	1	2	3	4
temp	Relative β-galactosidase levels			
23°C	1.00±0.000	1.05±0.004	1.14±0.06	1.04±0.02
37°C	1.23±0.03	1.52±0.09	1.667±0.38	2.10±0.03

Cells grown at 23°C in SD to 1×10^7^ cells/ml were transferred to fresh SD at 23°C or 37°C and assayed for β-galactosidase activity at the indicated times. The results represent the average data from three independent transformants. The results are relative to time 0 at 23°C. The strain tested was Y1890 carrying *UASru-3xAB-lacZ* integrated within genomic *LEU2*.

**Figure 4:**
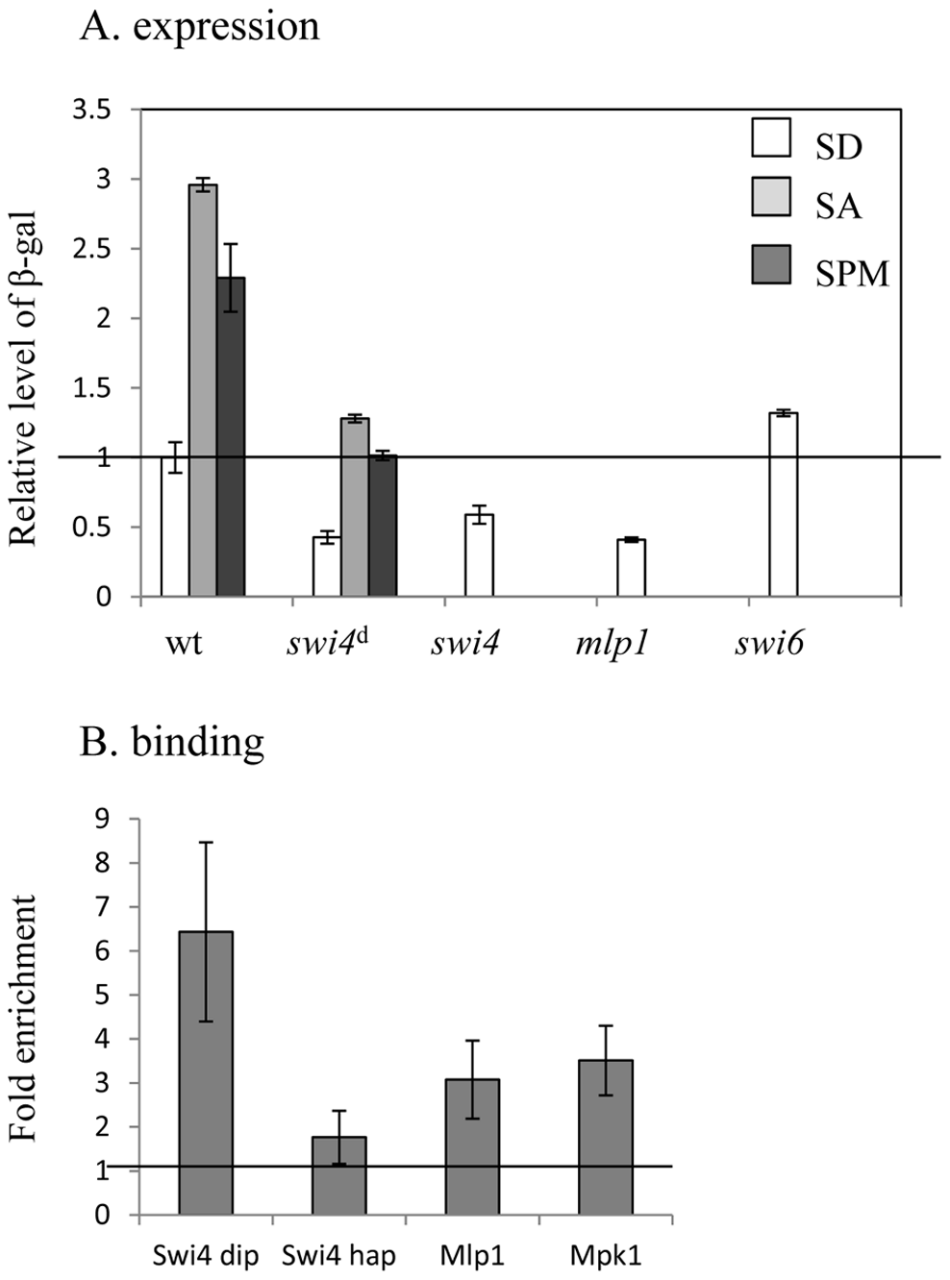
UASru is activated by binding to Swi4, Mlp1, or Mpk1. A. UASru activation. Cells were cultured at 37°C in SD, SA, and SPM as indicated in the figure. The β-galactosidase activity of the strains is expressed relative to wt cells grown in SD and represents the data from three independent transformants. Strains used were as follows: haploid wt Y1214 or Y1890 and diploid Y1721, *swi4*Δ (Y1855), *swi4*Δ/*swi4*Δ (*swi4* ^d^ –Y1912), *mlp1*Δ (Y1963), and *swi6*Δ (Y1958). These strains carried either *UASru-lacZ* (Y1214, Y1721, Y1855, and Y1912) or *UASru-3xAB-lacZ* (Y1890, Y1963 and Y1958) integrated within genomic *LEU2*. B. ChIP analysis of UASru binding. Cells were cultured in SD medium. The *UASru* element within the UASru-*lacZ* and *TEL1* were analyzed using qPCR. Binding is expressed as the ratio of UASru-*LacZ* to *TEL1* DNA normalized to the value of IPs performed without primary antibody. The results represent the averages of analyses of three independent colonies. Strains used were as follows: diploid Y1915 and haploid Y1928, carrying *SWI4-13myc* integrated within genomic *SWI4*. The haploid strains Y1961 and Y1962 carry genomic copies of *MLP1-6HA* or *MPK1-6HA*, respectively, and *UASru-lacZ* integrated within genomic *LEU2*.

The CWI pathway transmits its signal through either the transcriptional activator Rlm1 or through a complex between Swi4 and either MAPK Mpk1 or its pseudokinase paralog Mlp1 [43]. These two complexes bind to a Cell Cycle Box to which the Swi4/Swi6 (SBF) complex also binds [43], [44]. Deletion of *RLM1* did not detectably affect transcription of *UASru-GFP*. In contrast, deletion of *SWI4* significantly reduced its expression (Table 2), suggesting that the CWI pathway activates UASru through the Swi4/Mlp1, or Swi4/Mpk1, or both. Indeed, deletion of either *SWI4* or *MLP1* reduced the activity of UASru in haploid cells grown in SD (Fig. 4).

Deleting *SWI4* from diploid cells growing in SD, SA or SPM media (Fig. 4A) reduced reporter activity, demonstrating that Swi4 functions as a positive regulator of UASru under these growth conditions. Deleting Swi6 slightly but significantly increased the activity of UASru (Fig. 4A), indicating that the activity of UASru is regulated by the CWI signal and not by the cell-cycle SBF complex. We assume that the increase in transcription observed in *swi6*Δ cells might reflect loss of competition by the Swi4/Swi6 complex.

The sequence of UASru contains a putative binding site for Swi4 (CAgGAAA), which differs from the SBF consensus site at one position. This suggests that the Swi4/Mlp1 or Swi4/Mpk1 complexes bind to and directly activate this element, which is consistent with the results of the qChIP assay demonstrating specific binding of Swi4, Mlp1, and Mpk1 to UASru (Fig. 4B). We conclude, therefore, that temperature regulates the activity of UASru through the CWI pathway and that Swi4 binds to UASru in a complex with either Mlp1 or Mpk1.

### The Nitrogen Signal

**Figure 5:**
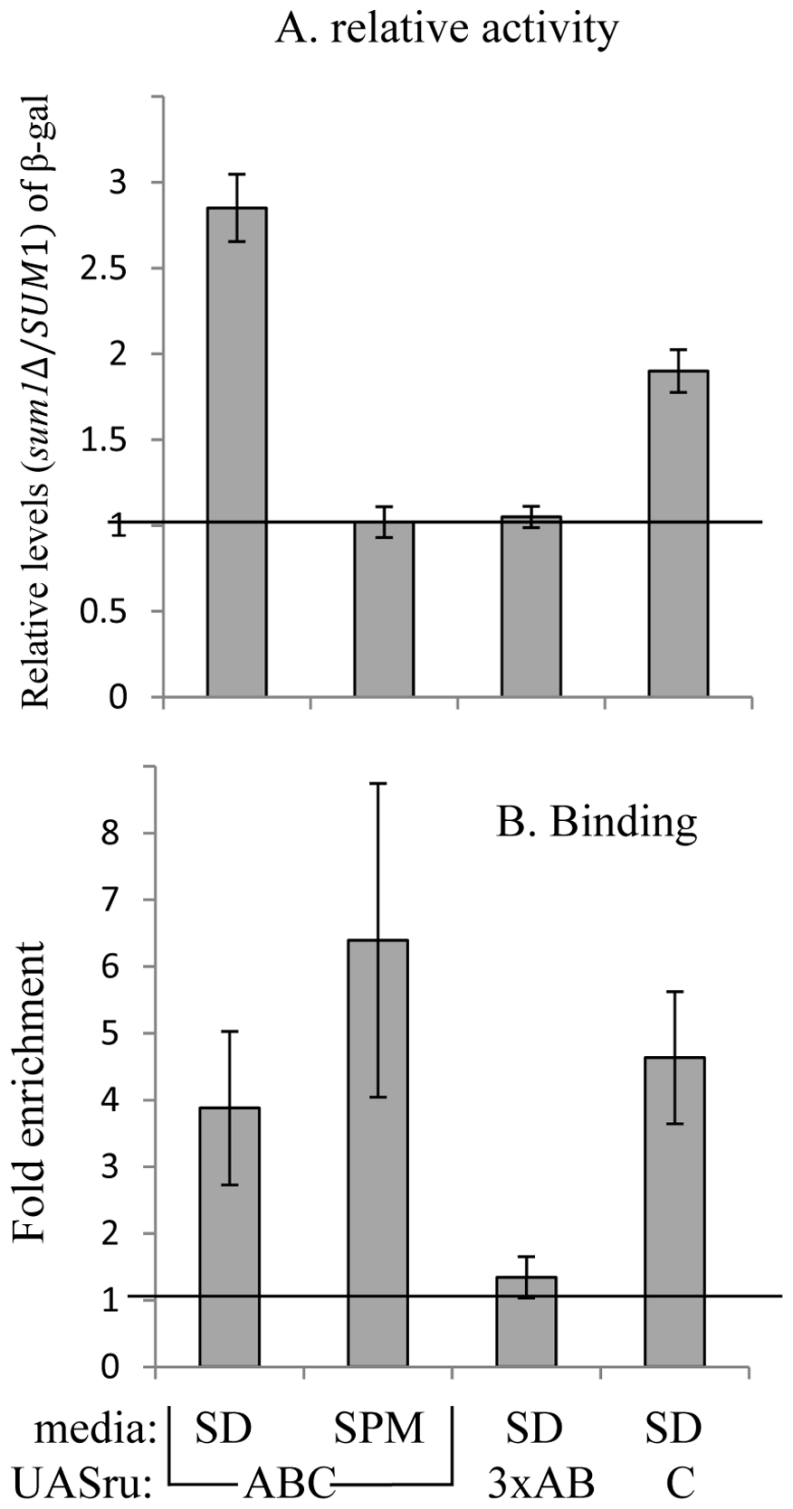
Sum1 represses the activity of UASru by binding to the C region. A. Analysis of β-galactosidase activity. Cells were cultured in SD medium, or after 6 h in SPM. Activation is expressed as the ratio of *sum1*Δ to *SUM1* β-galactosidase activity. The results represent the average for three independent transformants. Haploid strains used were as follows: wt (Y1214) and its isogenic strain *sum1*Δ (Y1827) carrying *UASru-lacZ*, wt (Y1890) and its isogenic strain *sum1*Δ (Y1953) carrying *UASru-3xAB-lacZ*, and wt (Y1743) and its isogenic strain *sum1*Δ (Y1959) carrying *UASru-C-lacZ*. B. ChIP analysis of UASru sub-elements. Cells were cultured in SD medium or in SPM for six hours. The amounts of *UASru* sub-element carried by the *lacZ* reporter genes and *TEL1* DNA were determined using qPCR. Binding is expressed as the ratio of sub-element to *TEL1* DNA normalized to the value of IPs without primary antibody. The results represent the data from analysis of three independent colonies. Haploid strains used are as follows: Y1837, Y1919, and Y1926 carrying SUM1-6HA as well as *UASru-lacZ*, *UASru-AB-lacZ*, and *UASru-C-lacZ*.

The transcription factor Sum1 binds to and represses the activity of UASru [20]. Here we show that the activity of Sum1 is mediated through UASru-C, because it binds to and represses reporter activity to a greater extent when compared with that of a UASru-AB (Fig. 5). Moreover, the only potential Sum1-binding site is present in UASru-C (GCCGCAAAg). Reporter-gene transcription was increased in *SUM1* deletion mutants cultured in SD but not in SPM (Fig. 5A), suggesting that repression of transcription by Sum1 is relieved in the absence of a nitrogen source. Moreover, Sum1 binds UASru in cells grown in either SD or SPM (Fig. 3B) and loses its activity in SPM in contrast to its ability to bind its recognition element.

### The Filamentation and Pheromone Pathways

**Figure 6:**
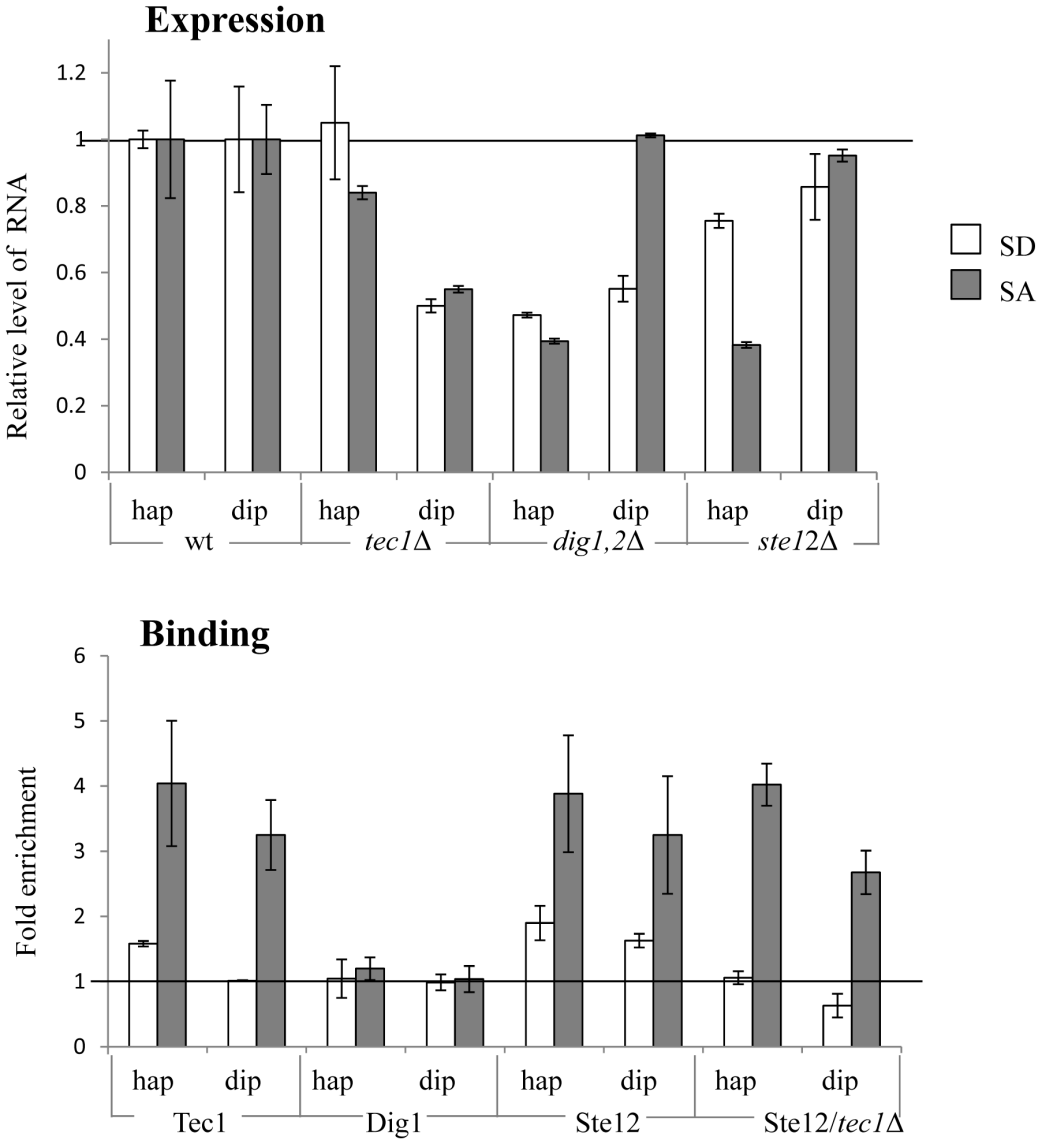
Direct and indirect activation of UASru by Ste12/Tec1 and Dig1/2, respectively. A. β-galactosidase assay. Cells were cultured in either SD or SA. The levels of enzyme activity relative to those of the wt isogenic strains are shown and represent the average data from three independent transformants. The isogenic haploid strains were as follows: wt (Y1214), *tec1*Δ (Y1963), *ste12*Δ (Y1627), *dig1*Δ *dig2*Δ (Y1647) and its isogenic wt haploid strain (Y1648). Diploid strains were as follows: *tec1*Δ/*tec1*Δ (Y2006) and its isogenic diploid strain (Y1721), *ste12*Δ/*ste12*Δ (Y1387) and its isogenic *ste12*Δ/*STE12* strain (Y1386), and *dig1*Δ *dig2*Δ/*dig1*Δ *dig2*Δ (Y1683) and its isogenic wt (Y1684). B. ChIP analysis. Cells were cultured in SD or SA. The levels of *UASru* (UASru-*lacZ* reporter) and the nonspecific *TEL1* DNAs were determined using qPCR. The data are expressed as the ratio of UASru to *TEL1* DNA normalized to the value of assays without primary antibody and represent the average data from three independent colonies. Strains used were as follows: Y2021 and Y2025 are haploid and diploid strains, respectively, carrying genomic *TEC1-13xmyc*; Y2003 and Y2005 are haploid and diploid strains, respectively, carrying genomic *DIG1-13xmyc*. Y1865 and Y2000 are haploid and diploid strains, respectively, carrying genomic *STE12-13xmyc*. Y2034 and Y2036 are haploid and diploid strains, respectively, carrying *tec1::HIS3* and *ste12::STE12-13myc-tADH1*. These strains also carried *UASru-lacZ* integrated within genomic *LEU2*.

In budding yeasts, nutrient depletion results in a dimorphic switch leading to pseudohyphal growth by diploid cells and invasion of a semi-solid matrix by haploid cells. This phenotype, which is mediated through a MAPK cascade, cannot be detected in the R-SGA screen, because deleting certain components causes sterility (reviewed in [5], [45]), and the insertion of the reporter genes to the array is through mating. We reasoned that this pathway may affect the activity of UASru, because the R-SGA screen revealed that deletion of its target TF, *TEC1*, reduced UASru activity ( [20] and Fig. 6A).

Because these pathways generate cell type-specific responses, we tested their relevant components in both haploid and in diploid cells. The results of qChIP analysis revealed that Tec1 associated with UASru mainly in cells grown in SA (Fig. 6B), suggesting that its effect was direct. Lack of binding in SD was not due to technical problems as efficient binding to the promoters of two known targets, *FUS1* and *CLN1,* was detected (data not shown). These results suggest that the effect of Tec1 in diploid cells grown in SD (Fig. 6A) was either indirect or that in SD, binding of an additional protein prevented detection of Tec1 binding. Deletion of *TEC1* in haploids caused a slight reduction in reporter gene expression in cells grown in SA, but none in SD (Fig. 6A). This result suggests that in haploid cells grown in SD, the activity of Tec1 may be inhibited by either posttranslational modification or active repression by the presence of an inhibitor/repressor.

Dig1 and Dig2 are transcriptional repressors that bind to Filamentation Responsive Element (FRE)-regulated genes, inhibit their expression [46],[47], and are negative regulators of Tec1. Therefore, deletion of *DIG1* and *DIG2* would be expected to increase the activity of UASru. In contrast, when both were deleted, the activity of UASru was decreased in cells grown in SD (Fig. 6A). In cells grown in SA, deletion of *DIG1* and *2* also reduced the expression of the reporter gene, but only in haploid cells (Fig. 6A). These results suggest that Dig1, Dig2, or both are positive activators in agreement with recent reports demonstrating that Dig2 also functions as a positive regulator of genes controlled by the Pheromone-Response Element (PRE) [48], [49] through stabilization of its associated TF, Ste12 [49]. Thus, the repression of gene expression by Dig2 depends on its binding to the target gene’s promoter. In contrast, activation does not require binding to DNA, because it affects the size of the pool of free Ste12 [49].

This hypothesis leads to two predictions as follows: (*i*) Dig1 and 2 will not bind UASru, because they function only as positive regulators; and (*ii*) Ste12 will also function as a positive regulator. The results of qChIP assays confirmed the first prediction, because Dig1 was not detected bound to UASru (Fig. 6B), although it bound the *FUS1* promoter in a control experiment (data not shown). Deletion of *STE12* caused a significant decrease in UASru activity in haploid cells (Fig. 6A), confirming the second prediction. We conclude, therefore, that the positive effect of Dig1 and 2 is indirect, likely through an effect on the level of Ste12. These results, however, cannot explain why Tec1 had no detectable effect in haploid cells grown in SD.

Tec1 binds to its target genes (FRE) as a heterodimer with Ste12 [50]. Consistent with these findings, Ste12 bound UASru in haploid or diploid cells grown in either SD or SA, yet binding efficiency was increased in SA (Fig. 6B). Unlike Tec1, the effect of Ste12 on UASru was more significant in haploid cells compared with diploid cells (Fig. 6A). A hypotheses to explain the ploidy effect is as follows: (*i*) The effect of Dig1 is more prominent in haploids, consequently the level of Ste12 is increased and the effect of deleting *STE12* is elevated; and (*ii*) in *MAT* **a**/*MAT*α diploids, the transcription of *STE12* is repressed [51] and the level of Ste12 is reduced 3-fold [52]. However, this possibility is inconsistent with the efficiency of binding of Ste12, because there was no significant difference between haploid and diploid cells (Fig. 6B).

Ste12 binds DNA either as a homodimer or heterodimer with Tec1 [50]. The inconsistent results with respect to the binding and transcriptional activation by Tec1 and Ste12 suggest that Ste12 may affect the activity of UASru without Tec1 and that Ste12 binding will occur in *TEC1* deletion mutants. The results of qChIP analysis revealed that, independent of ploidy, deletion of *TEC1* resulted in loss of Ste12 binding in SD but not in SA (Fig. 6B). These results suggest that Ste12 affects the activity of UASru through two mechanisms as follows: (*i*) In the presence of glucose, it binds UASru as a heterodimer with Tec1; and (*ii*) in the absence of glucose and the presence of acetate, it binds DNA as a homodimer. Nonetheless, our results cannot exclude the possibility that in SA it also binds as a heterodimer with Tec1.

The activities of Ste12/Ste12 and Tec1/Ste12 are regulated by mating and filamentation signals, respectively, which are transmitted through MAPK cascades that share many components [5]. The MAPK Kss1 activates FRE-regulated genes. In contrast, the MAPK Fus3 is the major activator of PRE-regulated genes. Moreover, Kss1 can transmit the mating signal to PRE- regulated genes, whereas Fus3 inhibits the activity of the FRE element by phosphorylating Tec1, causing the degradation of Tec1 by the proteasome [53]. We determined the effects of Kss1, Fus3, Tec1, and Ste7, which activate FRE- and PRE-regulated genes, on the activity of UASru [5].

**Figure 7:**
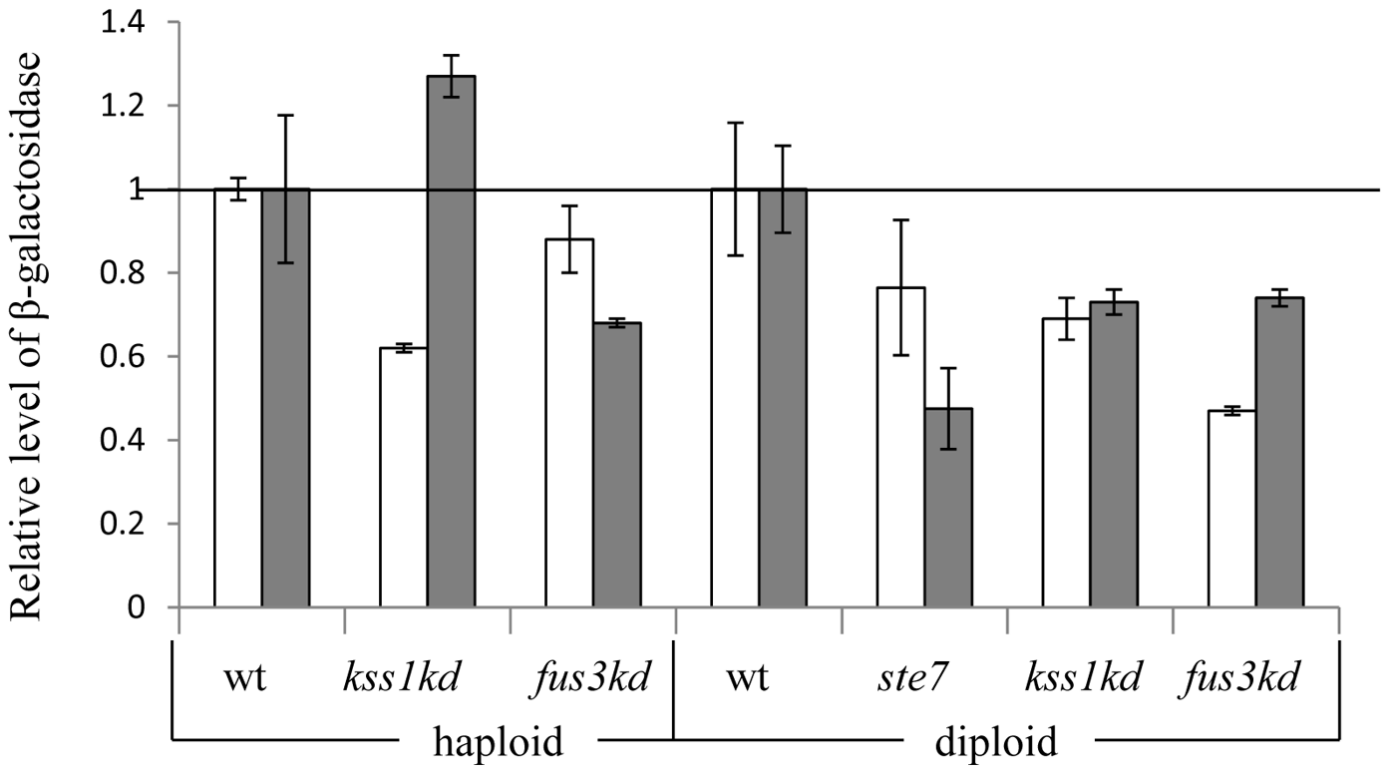
Fus3 and Kss1 regulate UASru activity. Cells were cultured in either SD or SA. The data are expressed as the level of β-galactosidase activity relative to that of the wt isogenic strain and represent the results from three independent transformants. The isogenic strains used were as follows: *kss1K42R* (Y1687), *fus3k42R* (Y1671) and their isogenic wt haploid strain (Y1214); *kss1K42R*/*kss1K42R* (Y1690), *fus3*Δ/*fus3k42R* (Y1678) and their isogenic wt diploid strain (Y1721); and *ste7*Δ/*ste7*Δ (Y1273) and its isogenic strain *ste7*Δ/*STE7* (Y1272). These strains also carried *UASru-lacZ* integrated into genomic *LEU2*.

In diploid cells, deletion of *STE7* and kinase-dead (kd) alleles of either *KSS1*or *FUS3* reduced the expression of *UASru-lacZ* (Fig. 7), suggesting that both MAPKs activate UASru. Haploid cells expressing Fus3-kd showed reduced expression of *UASru-lacZ* (Fig. 7), indicating that Fus3 is a positive regulator of UASru. Haploid cells expressing Kss1-kd showed reduced or increased activity of UASru when grown in SD or SA, respectively (Fig. 7). Because deletion of *TEC1* did not detectably affect UASru-mediated expression in haploids (Fig. 6A), Kss1 may act through a Ste12 homodimer. Further work is required to reveal why Kss1 inhibits, rather than activates as expected, in haploids grown in SA.

### UASru, FRE, and PRE are Distinct Elements that Respond to Specific Signals

The potential involvement of the Kss1 and Fus3 MAPK cascades in the regulation of UASru raises the question whether the filamentation and mating signals, which activate these kinases, will also activate UASru. Alternatively, does the signal that activates UASru (absence of glucose, with acetate as the sole carbon source with or without a nitrogen source) activate the FRE and/or PRE elements? To answer these questions, we determined the response of reporter genes regulated by UASru, FRE, or PRE elements in response to these signals.

The activity of UASru was not detectably modulated by addition of pheromone (Table 5). Moreover, the carbon and nitrogen sources that activate UASru inhibited the activity of the pheromone response element (PRE) (Table 7). We conclude, therefore, that yeast cells can use the same pathway to transmit specific signals to the PRE and UASru elements, which lead to mating or meiosis, respectively. The expression *FRE-lacZ* was induced in cells grown in SLAD, which contains a poor nitrogen source and glucose as the sole carbon source (Table 8). These conditions increased the expression of *UASru-lacZ* compared with that in cells grown in SD (Table 8). In contrast, nitrogen depletion in the absence of glucose (SPM) enhanced the induced expression of *UASru-lacZ* but had no effect on the activity of the FRE element (Table 8). These results indicate that filamentation and meiosis are alternative developmental pathways that respond to specific signals, although regulated by the same MAPK and TFs.

**Table 7:** Meiotic signals inhibit the function of the PRE element in response to α-factor.

Treatment	SD	SA	SPM 6 h
	β-galactosidase activity (Miller units)		
None	0.70±0.09	0.62±0.04	1.33±0.04
α-factor	110.78±4.35	14.86±0.07	2.65±0.09

The α-factor was added to a final concentration of 5 µg/ml for 3 h. The β-galactosidase activity represents data from three independent transformants. The strain tested was haploid wt Y1064 carrying PRE(*FUS1*pr)-*lacZ* (YCp1174).

**Table 8:** UASru and FRE elements respond specifically to meiotic and filamentation signals.

Reporter	SLAD	SD	SPM 6 h
	β-galactosidase activity (Miller units)		
UASru-*HIS4*-*lacZ*	24.75±1.89	32.87±1.28	117.77±3.78
*FRE-cyc1_TATA_ –lacZ*	17.21±3.58	2.83±0.19	3.16±0.78

The results represent the average of three independent transformants. Strains were as follows: Y1721 carrying *UASru-his4-lacZ* in the genome and Y422, which carry on the 2 μ plasmid *FRE(TY1*)-*cyc1-lacZ* (YEp2949).

## Discussion

In budding yeast, exit from the cell cycle and entry into meiosis depends on multiple signals, including mating type, absence of glucose, starvation, and stress. These signals are transmitted to the transcriptional activator *IME1*, which serves as the master regulator of meiosis (reviewed in [3], [9]).Transcriptional control is mediated through activators and repressors that bind to DNA, as well as through noncoding sense or antisense RNAs (ncRNAs) that may interfere in *cis* with the transcription of mRNA [54]–[56]. Indeed, the transcription of *IME1* is repressed in *MAT* **a** or *MAT*α haploids by the lncRNA *IRT1*, but not in *MAT* **a**/*MAT*α diploids, [17].

**Figure 8:**
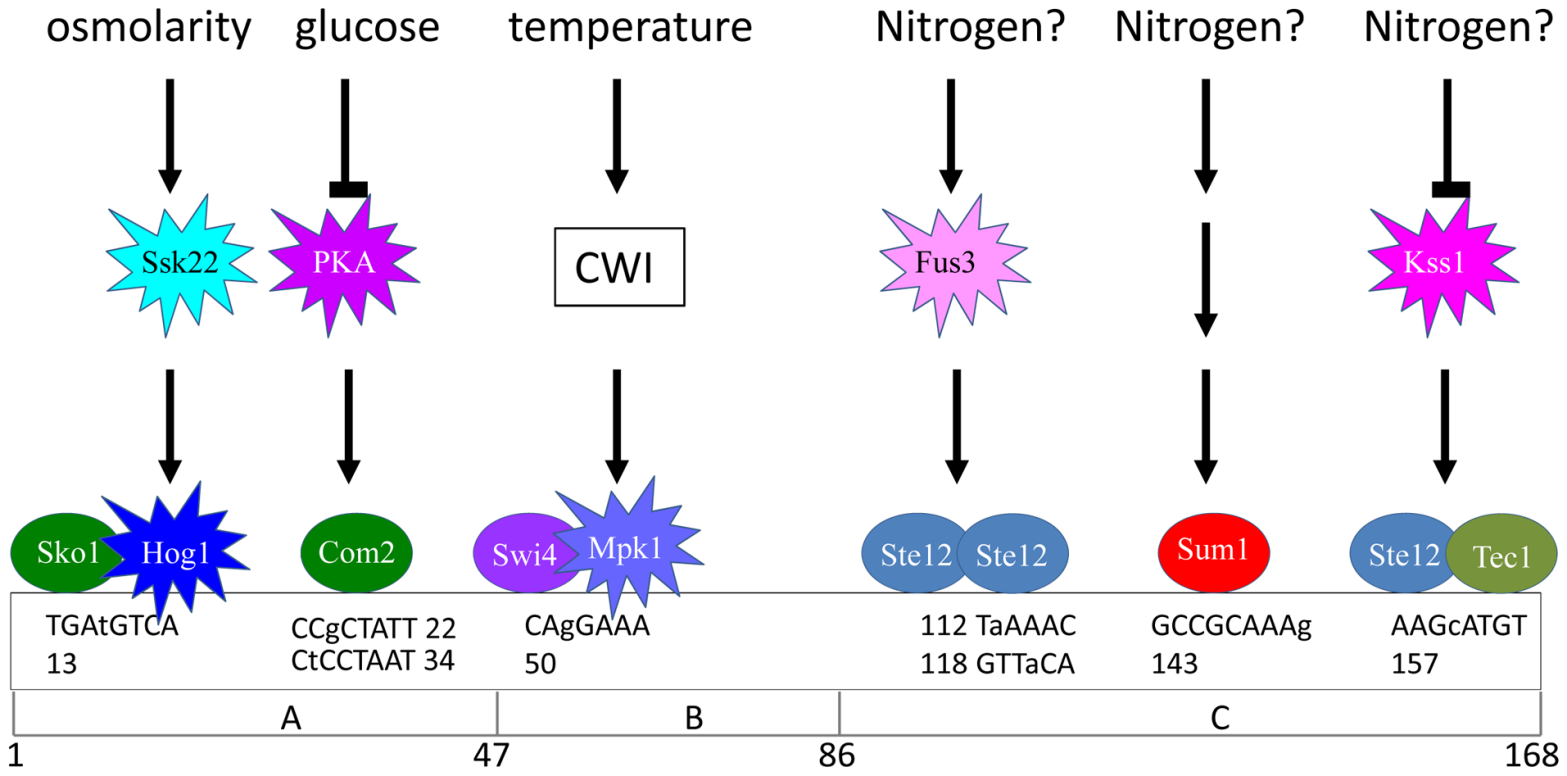
Summary of signaling events that influence the activity of the *IME1* UASru element. Putative TF recognitions sequences are shown above the diagram of UASru, and lower case letters indicate deviations from the consensus. Kinases, transcriptional activators, and repressors are indicated by stars and ovals, respectively.

The present study was aimed at identifying each of the transcription factors that control the activity of UASru, a specific region within the *IME1* promoter that when deleted, significantly reduces the transcription of *IME1* (Fig. 1 and Table 1). To identify the *cis*-acting factors required to regulate this region, we used reporter genes that are not subject to regulation by chromatin remodeling or inhibitory noncoding RNAs that may affect the binding of transcription factors. We show here that UASru activity is regulated by nutritional and stress signals, including glucose, nitrogen, osmolarity, and elevated temperature (Figs. 2 and 8, Tables 1, 4, 6). Moreover, these signals are transmitted by different pathways as follows: The glucose signal is transmitted by the cAMP/PKA pathway; the osmolarity signal by the Hog1 MAPK; the temperature signal by the CWI MAPK pathway; and the nitrogen signal likely by the filamentation (Kss1) and pheromone (Fus3) MAPK pathways as well as by Sum1 through an unidentified pathway. We assume that these signal pathways act independently of each other, all contributing to the response of *IME1* and meiosis to various stress and nutrient signals. The nutrient signals, i.e. glucose and nitrogen, also regulate additional elements within *IME1* promoter. Since deletion of these elements reduces the expression of *IME1* [9] and Fig. 1), we further suggest that these signals regulate the activity of UASru in an additive mode. Note that the only signal that prevents initiation of meiosis in diploids is the presence of glucose, whereas the level of Ime1 protein is not essential for efficient meiosis [57]. Consequently, any stress (rather than a combination of several signals) which induces the expression of Ime1 suffices for induction of meiosis. These findings are unique among yeast genes, because the UASru element is regulated by all known *S. cerevisiae* vegetative MAPKs, except for the spore wall assembly pathway in which the MAPK Smk1 is expressed only during meiosis [58]. This likely reflects Ime1’s role as a master regulator that is required for execution of gametogenesis.

### The Glucose Signal

In the presence of acetate as the sole carbon source, the activities of UASru (UASru-ABC) and UASru-AB were significantly increased, while the B region had no detectable UAS activity. We found that the cAMP/PKA pathway transmits this signal to UASru (Tables 2 and 3) and that Sko1 and Com2 directly repressed UASru activity (Tables 2 and 3). Sko1 functions as a transcriptional repressor that is phosphorylated by PKA, which inhibits its activity [59]. We show here that Sko1 binds to and represses the activity of UASru in cells grown in SD (Fig. 3 and Table 3). In the absence of glucose, the activity of PKA is reduced [38], and consequently the repression activity of Sko1 is also reduced (see below). We suggest that the repressor activity of Com2 depends on its phosphorylation by PKA, because it is phosphorylated in a domain that includes a PKA phosphorylation consensus motif [36]. Moreover, we show that alanine mutants of the serine residues of the PKA motif abolished the repressor activity of Com2 (Table 3). Thus, repression in the presence of glucose is transmitted by PKA to two targets, Sko1 and Com2, that affect the function of UASru-AB. Further, their respective putative binding sites are located in region A (Table 3 and Figs. 3 and 8).

We suggest that cAMP/PKA is not the only pathway that transmits the glucose signal, because deletions of its components did not completely abolish derepression in SD (Table 3). The Kss1 MAPK cascade may provide this function based on the observations as follows: (*i*) In haploid cells Kss1 activates invasive growth in response to glucose limitation [5]; (*ii*) the activity of UASru in haploid cells expressing a *kss1* kd allele was decreased in cells grown in SD and increased in cells grown in SA (Fig. 7); and (*iii*) Tec1 must bind UASru in cells grown in SD, because its deletion reduced binding of Ste12 (Fig. 6B). However, this possibility is incompatible with the observations as follows: (*i*) Deletion of *TEC1* did not affect activity in cells grown in SD and caused only a minor inhibitory effect in cells grown in SA; (*ii*) UASru-AB does not carry a putative binding site for Tec1/Ste12 or Ste12/Ste12; and (*iii*) Fus3 and Ste12 are more potent activators than Kss1 or Tec1 (Fig. 7). Therefore, the role of the Kss1, or Fus3, or both, in transmitting the glucose signal remains to be determined.

### The Osmotic Stress Pathway

We showed that in the presence of NaCl, UASru activity was increased independent of the carbon source (Table 4). This effect may be directly mediated through the Hog1 pathway based on the evidence as follows: (*i*) Deletion of Hog1 pathway components, including *HOG1*, significantly affected the activity of UASru (Tables 2 and 4); (*ii*) Hog1 binds to UASru depending on exposure to NaCl (Fig. 3). We suggest that osmotic regulation is mediated by the Sko1 repressor whose activity is abrogated by Hog1 phosphorylation [40], [59]. Thus, Sko1 binds UASru (Fig. 3), and *UASru-lacZ* expression is significantly increased in *SKO1* deletion mutants (Table 3). Sko1 may therefore serve as a node where signals transmitted through cAMP/PKA and Hog1 converge, leading us to propose that the direct repression of Sko1 on UASru activity in cells grown in SD is mediated by PKA. In contrast, repression is relieved by decreased PKA activity in cells grown in the absence of glucose and by activation of Hog1 in cells exposed to high osmolarity.

### The Temperature Signal

The level of *IME1* RNA is significantly increased following a shift to 37°C for 3 h [42]. Here we show that this effect is partially mediated by UASru (Table 6) through the CWI pathway, culminating in activating the transcription factor Swi4, which binds DNA as a heterodimer with either Mpk1 or its pseudokinase paralog Mlp1 (Table 2 and Fig. 4). These two complexes bind a Cell Cycle Box (CCB) [43], [44]. There is a CCB (one mismatch) within UASru region B (Fig. 8).The transcription factor Pog1 [60] binds to and activates the CCB. This TF was not present in our deletion array, and therefore was not identified. However, Pog1 binds the *IME1* promoter (−750 to −1050 from the RNA start site) and is required for the efficient *IME1* expression [17]. Because UASru resides within the Pog1-binding site, it is possible that Pog1 and Swi4/Mlp1 and Swi4/Mpk1 heterodimers bind to the same site.

### The Nitrogen Signal

UASru-C functions both as a URS and a UAS that are regulated by nitrogen. Repression is mediated by Sum1, which binds UASru-C under all growth conditions and represses UASru-C activity only in the presence of a nitrogen source (Fig. 5). Sum1 also represses the transcription of *NDT80* and certain mid meiosis-specific genes [61]. However, in this case relief of repression occurs when Sum1 dissociates from promoters upon phosphorylation by Ime2, Cdc28, and Cdc7 [62]–[64]. Binding of Sum1 to UASru was detected in haploid cells that do not express *IME2*, suggesting a specific mechanism that inhibits Sum1 activity, which depends on the Set3 histone deacetylase complex [65]. Using the R-SGA screen we detected enrichment of Set3 and complex components Hos2, Snt1, and Sif2 (data not shown), suggesting they act on UASru through Sum1.

The low UAS activity of UASru-C in the absence of a nitrogen source (Fig. 2) suggests that activity depends on a transcriptional activator. Candidates include Ste12/Tec1 and Ste12/Ste12, and the Kss1 and Fus3 MAPK pathways for the reasons as follows: (*i*) Nitrogen limitation in the presence of glucose activates the Kss1 MAPK pathway [5]; (*ii*) UASru activity is reduced in cells expressing a kd allele of either Kss1 or Fus3; (*iii*) Tec1 and Ste12, which activate the transcription of FRE-regulated genes, bind to and activate UASru (Fig. 6); (*iv*) Ste12 binds UASru in the absence of *TEC1*; and (*v*) an imperfect binding sites for Tec1 and Ste12 is present in UASru-C (Fig. 8).

### MAPK Specificity

MAPK pathways present in all eukaryotes transmit specific signals to diverse targets using shared components through an unknown mechanism. Here we show that UASru (meiosis), PRE (pheromone response), and FRE (filamentation response), share pathway components. UASru is activated in the absence of glucose but is not activated by either pheromones or nitrogen limitation in the presence of glucose (Tables 5, 6, 7, 8). PRE is not activated by glucose and nitrogen depletion (Table 7), and FRE, which is activated upon nitrogen limitation and the presence of glucose, is not activated when glucose is absent and nitrogen is depleted (Table 8). The pathways that transmit these signals and the transcription factors are identical. Thus, UASru is regulated by Fus3 and Ste12/Ste12 (Figs. 6, 7, 8), which activate PRE [5] as well as by the Kss1 MAPK cascade and Ste12/Tec1 (Figs. 6, 7, 8), which activate FRE [5].

Therefore, our results raise additional questions regarding signaling specificity. The *IME1* (or only UASru) and *FLO11* (required for filamentation) carry responsive elements for both the MAPK and cAMP/PKA pathways (Fig. 8, Table 3, and [11], [31] [9]) that regulate these genes oppositely. Thus, glucose and PKA repress the activity of UASru (Tables 2, 3) but activate *FLO11* transcription [31]. This may explain specificity and why filamentation and meiosis (expression of *IME1*) represent alternative developmental pathways. Thus, repression of UASru by PKA prevents activation by Kss1 and Tec1/Ste12, allowing UASru to specifically respond to the meiotic signal. However, we used a FRE-driven reporter gene that includes only the binding site for Tec1/Ste12, and, therefore, this mechanism does not apply to the lack of response of FRE to the meiotic signal.

## Supporting Information

Table S1 List of plasmids.
(DOCX)

Table S2 List of strains.
(DOCX)

Table S3 List of oligonucleotides.
(DOCX)
