# Supplementary material for: Multiple MAPK Cascades Regulate the Transcription of IME1, the Master Transcriptional Activator of Meiosis in Saccharomyces cerevisiae
Source: PLoS One. 2013 Nov 13;8(11):e78920. doi: 10.1371/journal.pone.0078920 (PMC3827324; doi:10.1371/journal.pone.0078920)
Supplement: Table S1 — List of plasmids. (DOCX) [file pone.0078920.s001.docx]

TABLE 1S. List of plasmids

| **Name** | **details** | **Remarks, reference** |
| --- | --- | --- |
| YCp117 | *HO* in YCp50 | B495, G. Fink |
| YCp1174 | *FUS1-LacZ* in YCp50 | B1497, G. Fink |
| YIp1267 | *swi4::URA3* | 1636, K. Nasmyth |
| YIp1268 | *swi6::TRP1* | C1313, K. Nasmyth |
| YIp1408 | *ime1::URA3* (carries *IME1* deletion from  -1118 to +946) | our lab |
| YIp1722 | *MET3p-CLN2*, *TRP1* | SB232, A. Amon |
| YIp2007 | *his4-LacZ* , *LEU2* | Our lab ([Neiman 2011](#_ENREF_2); [Sagee *et al.* 1998](#_ENREF_3)) |
| YIp2025 | *ste12::URA3* | pNC163, B. Errede |
| YIp2029 | *fus3::TRP1* | B2069, J. Brill and G. Fink |
| YIp2033 | *ste7::URA3* | B2697, J. Brill and G. Fink |
| YIp2102 | *IME1-UASru-his4-LacZ, LEU2* | our lab |
| YIp2247 | *ime1(-3762 to +202)-lacZ, URA3* | our lab |
| YIp2389 | *tec1::HIS3* (deletion of amino acids 12 to 319) | B3662, G. Fink |
| YIp2643 | *kss1::hisG-URA3-hisG* | B3671, G. Fink |
| YIp2863 | *loxp-TRP1-loxp* | our lab |
| YIp2926 | *ime1ΔUASru (-4401 to –1364 and –1197 to +201)-lacZ, URA3* | this work |
| YIp2930 | *UASru(BC)-HIS4-lacZ, LEU2* | this work |
| YEp2949 | *FRE(TY1)-cyc1TATA-lacZ*, 2μ, *URA3* | BHUM0212, H. Madhani |
| YIp2958 | *3xUASru(AB)-his4-lacZ, LEU2* | this work |
| YIp2972 | *fus3K42R, HIS3* | this work |
| YIp2974 | *UASru(B) B-his4-lacZ, LEU2* | this work |
| YIp2975 | *UASru(AB)- his4-lacZ, LEU2* | this work |
| YIp2987 | *kss1-K42R, TRP1* | this work |
| YIp2988 | *UASru(C)-his4-lacZ, LEU2* | this work |
| P2999 | *IME1-ΔUASru* (IME1 is from -3.7 to +2.2) | this work |
| YIp3131 | *SUM1(+1782 to 3168)-6HA, k1TRP1* | this work |
| YIp3153 | *Sum1*Δ::*URA3* | this work |
| YIp3211 | *ste12(+486 to +2063)- 13myc-tADH1, TRP1* | this work |
| YIp3218 | *swi4(+1870 to +3278)-13myc-tADH1, TRP1* | this work |
| YIp3304 | *COM2-5’-6xHA-COM2-COM2-3’, TRP1* | this work |
| YIp3255 | *MLP1-5’-MLP1-6HA, k1TRP1* | this work |
| YIp3257 | *MPK1-5’-MPK1-6HA, k1TRP1* | this work |
| YIp3258 | *mlp1Δ::URA3gb* | this work |
| YIp3267 | *com2Δ::URA3gb* | this work |
| YIp3289 | *DIG1(+432 to +1355)-13myc-tADH1, TRP1* | this work |
| YIp3294 | *UAShis4-his4-LacZ, LEU2* | X-1 derivative ([Nagawa and Fink 1985](#_ENREF_1)) |
| YIp3296 | *UASru(C)-UAShis4-his4-lacZ, LEU2* | this work |
| YIp3299 | *TEC1(+14 to +1436)-13myc-tADH1, HIS3* | this work |
| YIp3310 | *COM2(-796 to +1311)-13xmyc-ADHt*, *HIS3* | this work |
| YIp3314 | c*om2S164AS88A*(+51 to 3’), *HIS3* | This work |

Nagawa, F., and G. R. Fink, 1985 The relationship between the "TATA" sequence and transcription initiation sites at the HIS4 gene of Saccharomyces cerevisiae. Proc Natl Acad Sci U S A **82:** 8557-8561.

Neiman, A. M., 2011 Sporulation in the Budding Yeast Saccharomyces cerevisiae. Genetics **189:** 737-765.

Sagee, S., A. Sherman, G. Shenhar, K. Robzyk, N. Ben-Doy *et al.*, 1998 Multiple and distinct activation and repression sequences mediate the regulated transcription of *IME1*, a transcriptional activator of meiosis- specific genes in *Saccharomyces cerevisiae*. Mol Cell Biol **18:** 1985-1995.
