# Supplementary material for: Multiple MAPK Cascades Regulate the Transcription of IME1, the Master Transcriptional Activator of Meiosis in Saccharomyces cerevisiae
Source: PLoS One. 2013 Nov 13;8(11):e78920. doi: 10.1371/journal.pone.0078920 (PMC3827324; doi:10.1371/journal.pone.0078920)
Supplement: Table S2 — List of strains. (DOCX) [file pone.0078920.s002.docx]

TABLE 2S. List of strains

|  | **Relevant genotype** | **Remarks Reference** |
| --- | --- | --- |
| Y422 | *MAT*a/*MATα, ura3-52/ura3-52, trp1*Δ*/trp1*Δ*, leu2-3,112/leu2-3,112, ade2-1/ade2-R8,*  *his4-519/HIS4, his6-1/HIS6, can1/CAN1* | Y546 x Y547 |
| Y422-R | *MAT*a/*MATα, leu2-3,112/leu2-3,112::LEU2-his4-lacZ* | Y422 derivative using YIp2007 |
| Y546 | *MAT*a, *ura3-52, trp1Δ, leu2-3,112, ade2-1, his4-519* | parent of Y1064 |
| Y547 | *MAT*α, u*ra3-52, trp1Δ, leu2-3,112, ade2-R8, his6-1, can^r^* | parent of Y1061 |
| Y1061 | *MAT*α, *ura3-52, trp1*Δ*, leu2-3,112, his3::URA3gb, ade2-R8, gal80::hisG* | derivative of Y547 |
| Y1062 | *MAT*α*, ura3-52, trp1*Δ*, leu2-3,112, his3::hisG, ade2-R8, gal80::hisG* | parent of Y1061 |
| Y1064 | *MAT*a*, ura3-52, leu2,3-112, trp1*Δ*, his3::hisG, ade2-1, met, gal80::hisG, gal4::hisG* | derivative of Y546 |
| Y1065 | *MAT*α*, ura3-52, trp1Δ, leu2-3,112, his3::hisG, ade2-R8, gal80::hisG, gal4::hisG* | derivative of Y1062 |
| Y1075 | *MAT*a*, ime1::hisG* | Y1064 derivative using YIp1408 |
| Y1092 | *MAT*a*, ste12::URA3* | Y1064 derivative using YIp2025 |
| Y1106 | *MAT*a*, ste7::URA3* | Y1064 derivative using YIp2033 |
| Y1139 | *MAT*α*, ura3-52, trp1-289, his3-1, leu2-3* | CEN.PK2α |
| Y1188 | *MAT*α*, ste7::URA3* | Y1065 derivative using YIp2033 |
| Y1214 | *MAT*a*, leu2,3-112::LEU2-UASru-his4-lacZ* | Y1064 derivative using YIp2102 |
| Y1264 | *MAT*a*, ste7::URA3, leu2,3-112::LEU2-IME1-UASru-his4-LacZ* | Y1106 derivative using YIp2102 |
| Y1265 | *MATa/MATα, ura3/ura3, trp1/trp1, his3/his3, leu2/leu2::LEU2-IME1-UASru-his4-LacZ* | CEN.PK2a/α background Eurofan |
| Y1267 | *gpr1*Δ/*gpr1*Δ, *leu2-3,112/leu2,3-112::LEU2-UASru-his4-LacZ* | Y1265 derivative |
| Y1270 | *ras2Δ::kanL/ ras2Δ::kanL, , leu2-3,112/leu2,3-112::LEU2-UASru-his4-LacZ* | Y1265 derivative |
| Y1272 | *MAT*a*/MAT*α*,leu2/leu2,3-112::LEU2-IME1-UASru-his4-LacZ, ste7::URA3-loxp/STE7* | Y1264 x Y1139 |
| Y1273 | *MAT*a*/MAT*α*, ste7::URA3-loxp/ste7::URA3-loxp, leu2-3,112/leu2,3-112::LEU2-UASru-his4-LacZ* | Y1264 X Y1188 |
| Y1338-2 | *cdc25Δ::lox-kan-lox/cdc25Δ::lox-kan-lox leu2-3/leu2-3::LEU2-IREu-his4-lacZ ura3-52/ura3-52::URA3-cdc25(145 to 1589)* | Y1265 derivative |
| Y1369 | *MAT*a*, leu2,3-112::LEU2-UASru-his4-lacZ,*  *ste12::URA3* | Y1092 derivative using YIp2025 |
| Y1371 | *MAT*α*, ste12::URA3* | Y547 derivative using YIp2025 |
| Y1386 | *MAT*a*/MAT****α****, ste12::URA3/STE12, leu2,3-112/leu2,3-112::LEU2-UASru-his4-lacZ* | Y1369 X Y547 |
| Y1387 | *MAT*a*/MAT*α*, ste12::URA3/ste12::URA3, leu2,3-112/leu2,3-112::LEU2-UASru-his4-lacZ* | Y1369 X Y1371 |
| Y1396 | *MAT*α*, fus3Δ::TRP1* | Y1062 derivative using YIp2029 |
| Y1623 | *MAT*a*/MAT*α*, ura3-52/ura3-52::URA3-ime1(-3762 to +202)-lacZ* | Y422 derivative using YIp2247 |
| Y1624 | *MAT*a*/MAT*α*, ura3-52/ura3-52::URA3 + ime1(-4401 to –1364 and –1197 to +201)-lacZ(*Δ*UASru)* | Y422 derivative using YIp2926 |
| Y1625 | *MAT*a*/MAT*α*, leu2-3,112/leu2-3,112::LEU2-*  *UASru(IME1 from 1318-1118)-his4-lacZ* | Y422 derivative using YIp2930 |
| Y1627 | *MAT*a*, ste12::URA3, leu2,3-112::LEU2-UASru-his4-lacZ* | Y1214 derivative using YIp2025 |
| Y1639 | *MAT*a*/MATα, ime1::hisG/IME1* | Y1065 x Y1075 |
| Y1645 | *dig1::HIS3, dig2::TRP1 ade2-101och, leu2-Δ1, his3-Δ200, lys2-801a, trp1-Δ63, ura3-52* | Y1065 derivative, JCY5, J. Thorner, |
| Y1646 | *ade2-101och, leu2-Δ1, his3-Δ200, lys2-801a, trp1-Δ63, ura3-52* | YPH499 J. Thorner |
| Y1647 | *dig1::HIS3, dig2::TRP1, leu2-Δ1:: LEU2-IME1-UASru-his4-LacZ* | Y1645 derivative using YIp2102 |
| Y1648 | *leu2-Δ1:: LEU2-IME1-UASru-his4-LacZ* | Y1646 derivative using YIp2102 |
| Y1670 | *MAT*a*/MAT*α*, leu2-3,112/leu2-3,112:: LEU2-UASru(AB)-his4-LacZ* | Y422 derivative using YIp2975 |
| Y1671 | MATa*, leu2,3-112::LEU2-UASru-his4-lacZ*,  *fus3K42R-HIS3* | Y1214 derivative using YIp2972 |
| Y1678 | *MAT*a/*MAT*α, *fus3K42R-HIS3/fus3Δ::TRP1* | Y1671 x Y1396 |
| Y1683 | *MAT*a*/MAT*α*, dig1::HIS3/dig1::HIS3, dig2::TRP1/dig2::TRP1, leu2-Δ1:: LEU2-IME1-UASru-his4-lacZ/leu2-Δ1:: LEU2-UASru-his4-lacZ* | Y1647 derivative using YCp117 |
| Y1684 | *MAT*a/*MAT*α, *leu2-Δ1:: LEU2-IME1-UASru-his4-LacZ/leu2-Δ1:: LEU2-IME1-UASru-his4-LacZ* | Y1648 derivative using YCp117 |
| Y1685 | *MAT*a*/MAT*α*, leu2-3,112/leu2-3,112::LEU2-IME1-UASru-his4-LacZ* | Y422 derivative using YIp2102 |
| Y1686 | *MAT*a*/MAT*α*, leu2-3,112/leu2-3,112::LEU2-3xUASru(AB)- his4-LacZ* | Y422 derivative using YIp2958 |
| Y1687 | *MAT*a*, kss1-K42R-TRP1, leu2,3-112::LEU2-UASru-his4-lacZ* | Y1214 derivative using YIp2987 |
| Y1688 | *MAT*α*, kss1-K42R-TRP1* | Y1061 derivative using YIp2987 |
| Y1690 | *MAT*a*/MAT*α*, kss1-K42R-TRP1/kss1-K42R-TRP1, leu2-3,112/leu2,3-112::LEU2-UASru-his4-lacZ* | Y1687 x Y1688 |
| Y1697 | *MAT*a*/MAT*α*, ura3-52/ura3-52, leu2-3,112/leu2-3,112:: LEU2-UASru(C)-his4-LacZ* | Y422 derivative using YIp2988 |
| Y1703 | *MAT*a*, kss1::hisG* | Y1214 derivative using Yip2643, following selection on 5FOA. |
| Y1721 | *MAT*a*/MAT*α*, leu2-3,112/leu2,3-112::LEU2-UASru-his4-lacZ* | Y1214 x Y1061 |
| Y1737 | *MAT*α*, ime1*Δ*UASru* | Y1649 derivative using P2999 and pRS423 |
| Y1741 | *MAT****a****/MATα, ime1*Δ*UASru/ime1::hisG* | Y1737 x Y1075 |
| Y1743 | *MAT*a, *leu2,3-112::LEU2-UASru-C-his4-LacZ* | Y1064 derivative using YIp2988 |
| Y1827 | MATa, *leu2,3-112::LEU2-UASru-his4-lacZ, sum1*Δ*::URA3* | Y1214 derivative using YIp3153 |
| Y1837 | *MAT*a*, Sum1-6HA, , leu2,3-112::LEU2-UASru-his4-lacZ* | Y1214 derivative using YIp3131 |
| Y1855 | *MAT*a*, swi4::URA3, leu2,3-112::LEU2-UASru-his4-lacZ* | Y1214 derivative using YIp1267 |
| Y1858 | *MAT*a*, tec1::HIS3, leu2,3-112::LEU2-UASru-his4-lacZ* | Y1214 derivative using YIp2389 |
| Y1862 | *MAT*a*, leu2,3-112::LEU2-UASru-his4-lacZ, hog1::TRP1* | Y1214 derivative using YIp2863 |
| Y1865 | *MAT*a*, leu2,3-112::LEU2-UASru-his4-lacZ, STE12-13myc-tADH1-TRP1* | Y1214 derivative using YIp3211 |
| Y1887 | *MAT*a*, SWI4-13xmyc, leu2,3-112::LEU2-UASru-his4-lacZ* | Y1214 derivative using YIp3218 |
| Y1889 | *MAT*α*, hog1*Δ*::TRP1* | Y1065 derivative using YIp2863 |
| Y1890 | *MAT*a*, leu2,3-112::LEU2-3xUASru(AB)- his4-lacZ* | Y1064 derivative using YIp2958 |
| Y1895 | *MAT*α*, swi4::URA3* | Y1065 derivative using YIp1267 |
| Y1907 | *MAT*α*, kss1::hisG-URA3-hisG* | Y1065 derivative using YIp2643 |
| Y1908 | *MAT*a*/MAT*α*, leu2-3,112/leu2,3-112::LEU2-UASru-his4-lacZ, hog1*Δ*::TRP1/hog1*Δ*::TRP1* | Y1862 x Y1889 |
| Y1909 | *MAT*a*/MAT*α*, kss1::hisG-URA3-hisG/kss1::hisG* | Y1907 x Y1703 |
| Y1911 | *MAT*α*, swi4::URA3, trp1*Δ*::TRP1-MET3p-CLN2* | Y1895 derivative using YIp1722 |
| Y1912 | *MAT*a*/MAT*α*, swi4::URA3/swi4::URA3, trp1Δ/trp1Δ::TRP1-MET3p-CLN2, leu2,3-112::LEU2-UASru-his4-lacZ/leu2-3,112* | Y1855 x Y1911 |
| Y1915 | *MAT*a*/MAT*α*, swi4::URA3/SWI4-13xmyc, leu2,3-112::LEU2-UASru-his4-lacZ/leu2-3,112, trp1Δ::TRP1-MET3p-CLN2/ trp1Δ* | Y1887 x Y1911 |
| Y1919 | *MAT*a*, SUM1-6HA-k1TRP1-sum1ΔN’ leu2,3-112-LEU2-IME1-UASru-c-his4-LacZ* | Y1743 derivative using YIp3131 |
| Y1924 | *MAT*a*, leu2-3,112::LEU2-UASruAB-his4-lacZ* | Y1064 derivative using YIp2975 |
| Y1926 | *MAT*a*, SUM1-6HA-k1TRP1-sum1ΔN’, leu2-3,112::LEU2-UASruAB-his4-lacZ* | Y1924 derivative using YIp3131 |
| Y1928 | *MAT*a,*, leu2-3,112::LEU2-UASruAB-his4-lacZ, swi4(+1870 to +3278)-TRP1-SWI4-13myc-tADH1* | Y1924 derivative using YIp3218 |
| Y1953 | *MAT*a, *sum1::URA3, leu2,3-112::LEU2-3x[UASru-AB]- his4-LacZ* | Y1890 derivative using YIp3153 |
| Y1958 | *MAT*a*, swi6::TRP1, leu2,3-112::LEU2-3xUASru(AB)- his4-LacZ* | Y1890 derivative using YIp1268 |
| Y1959 | MATa, leu2,3-112-LEU2-IME1-UASru-C-his4-LacZ, sum1::URA3 | Y1743 derivative using YIp3153 |
| Y1961 | *MAT*a*, MLP1::MLP1-6HA- k1TRP1, leu2,3-112::LEU2-UASru-his4-lacZ* | Y1214 derivative using YIp3255 |
| Y1962 | *MAT*a*, MPK1::MPK1-6HA-k1TRP1, leu2,3-112::LEU2-UASru-his4-lacZ* | Y1214 derivative using YIp3257 |
| Y1963 | *MAT*a*, mlp1-5’-URA3gb-mlp1, leu2,3-112::LEU2-3xUASru(AB)-his4-LacZ* | Y1890 derivative using YIp3258 |
| Y1968 | *MAT*a*, com2Δ::URA3gb, leu2,3-112::LEU2-3xUASru(AB)- his4-LacZ* | Y1890 derivative using YIp3267 |
| Y1987 | *MAT*a*, leu2,3-112::LEU2-UASru-his4-lacZ, com2*Δ::*URA3::hisG*,  *SWI4-13xmyc* | Y1887 derivative using YIp3267 |
| Y2000 | *MAT*a/*MAT*α, *leu2-3,112/leu2,3-112::LEU2-UASru-his4-lacZ*, *STE12/STE12-13myc-tADH1-TRP1* | Y1865 x Y1065 |
| Y2001 | *MAT*α*, tec1Δ::HIS3* | Y1065 derivative using YIp2389 |
| Y2003 | *MAT*a*, DIG1-13myc-tADH1-TRP1-dig1, leu2,3-112::LEU2-UASru-his4-lacZ* | Y1214 derivative using YIp3289 |
| Y2005 | *MAT*a*/MAT*α*, DIG1-13myc-tADH1-TRP1-dig1/DIG1, leu2,3-112::LEU2-UASru-his4-lacZ/leu2-3,112* | Y2003 x Y1065 |
| Y2006 | *MAT*a*/MAT*α*, tec1::HIS3/tec1::HIS3, leu2,3-112::LEU2-UASru-his4-lacZ/leu2-3,112* | Y1858 x Y2001 |
| Y2008 | *MAT*a, *leu2,3-112::LEU2-UASru-his4-lacZ, TEC1-13xmyc-HIS3* | Y1214 derivative using YIp3299 |
| Y2010 | *MAT*a, *leu2,3-112:: LEU2-* *UAShis4-his4-LacZ* | Y1064 derivative using YIp3294 |
| Y2011 | *MAT*a, *leu2,3-112:: LEU2- UASru(C)-UAShis4-his4-lacZ* | Y1064 derivative using YIp3296 |
| Y2018 | *MAT*α, *sum1::SUM1-6HA-k1TRP1* | Y1065 derivative using YIp3131 |
| Y2019 | *MAT*α, *tec1*Δ*::HIS3*, *com2*Δ*::URA3gb* | Y2001 derivative using YIp3267 |
| Y2021 | *MAT*a, *leu2,3-112::LEU2-UASru-his4-lacZ*, *TEC1-13xmyc-HIS3, COM2-5’-6xHA-COM2-COM2-3’- TRP1* | Y2008 derivative using YIp3304 |
| Y2025 | *MAT*a/ *MAT*α, *tec1*Δ*::HIS3/* *TEC1-13xmyc-HIS3*, *com2*Δ::*URA3gb/COM2, leu2-3,112/leu2,3-112::LEU2-UASru-his4-lacZ* | Y2019 x Y2021 |
| Y2028 | *MAT*a/*MAT*α, *leu2-3,112/leu2,3-112:: LEU2-* *UAShis4-his4-LacZ* | Y1065 x Y2010 |
| Y2029 | *MAT*a/*MAT*α, *leu2-3,112/leu2,3-112:: LEU2-* UASru-c-*UAShis4-his4-LacZ* | Y1065 x Y2011 |
| Y2032 | *MAT*a, *his3-*1Δ, *leu2-0*Δ, *ura3-0*Δ , *HOG1-HA::HIS3* | BY4741, F. Possas |
| Y2034 | *MAT*a, *tec1::HIS3*, *STE12- 13myc-tADH1*, *leu2,3-112::LEU2-UASru-his4-lacZ* | Y1858 derivative using YIp3211 |
| Y2035 | *MAT*α, *tec1Δ::HIS3, ste12::STE12- 13myc-tADH1* | Y2001 derivative using YIp3211 |
| Y2036 | *MAT*a/*MAT*α, *tec1*Δ::*HIS3*/*tec1Δ*::*HIS3*, *STE12- 13myc-tADH1/STE12- 13myc-tADH1, leu2,3-112::LEU2-UASru-his4-lacZ/leu2-3,112* | Y2034 x Y2035 |
| Y2037 | *MAT*a, *COM2-13xmyc-ADHt* | Y1214 derivative using YIp3310 |
| Y2045 | *MAT*a, *ura3*, *leu2*, *trp1*, *his3*, *ade2*, *3xHA-SKO1* | MAP37, F. Possas |
| Y2051 | *MAT*a, *com*2S164AS88A-*HIS3*,  *leu2,3-112::LEU2-3xUASru(AB)- his4-lacZ* | Y1890 derivative using YIp3314 |
| Y2052 | *MAT*a/*MAT*α, *com*2S164AS88A/*com2*Δ  *leu2,3-112::LEU2-3xUASru(AB)- his4-lacZ/ leu2-3,112* | Y2051 x Y2019 |
| Y2063 | *MAT*a, *leu2,3-112::LEU2-UASru-his4-lacZ*, *sko1Δ::lox-HIS3* | Y1214 derivative using a PCR one step deletion protocol ([Guldener *et al.* 1996](#_ENREF_1)) |

Guldener, U., S. Heck, T. Fielder, J. Beinhauer and J. H. Hegemann, 1996 A new efficient gene disruption cassette for repeated use in budding yeast. Nucleic Acids Res **24:** 2519-2524.

Nagawa, F., and G. R. Fink, 1985 The relationship between the "TATA" sequence and transcription initiation sites at the HIS4 gene of Saccharomyces cerevisiae. Proc Natl Acad Sci U S A **82:** 8557-8561.

Neiman, A. M., 2011 Sporulation in the Budding Yeast Saccharomyces cerevisiae. Genetics **189:** 737-765.

Sagee, S., A. Sherman, G. Shenhar, K. Robzyk, N. Ben-Doy *et al.*, 1998 Multiple and distinct activation and repression sequences mediate the regulated transcription of *IME1*, a transcriptional activator of meiosis- specific genes in *Saccharomyces cerevisiae*. Mol Cell Biol **18:** 1985-1995.
