# Supplementary material for: Multiple MAPK Cascades Regulate the Transcription of IME1, the Master Transcriptional Activator of Meiosis in Saccharomyces cerevisiae
Source: PLoS One. 2013 Nov 13;8(11):e78920. doi: 10.1371/journal.pone.0078920 (PMC3827324; doi:10.1371/journal.pone.0078920)
Supplement: Table S3 — List of oligonucleotides. (DOCX) [file pone.0078920.s003.docx]

Table 3S. List of oligonucleotides

| Amplified gene | Forward primer | Reverse primer |
| --- | --- | --- |
| UASru | CGTTGATGTCATCCGCTATT | CATACCTCGACATCACATGCT |
| UASru-AB | ACAGCGCAGTTGTGCTATG | CACAGGAAACAGCTATGACCA |
| UASru-C | AAATGCGGTAGCAAAGGAC | AACACACATCGGAGGTGAATA |
| *POL1* | GCTGATGCACCAGTTAATTCT | CACCCTGATCCACCTCTGAA |
| *TEL1* | GCGTAACAAAGCCATAATGCCTCC | CTCGTTAGGATCACGTTCGAATCC |
| *FUS1* | GCCACTCTTACATTGTCA | GATGCCCTTTTTGACGTA |
| *CLN1* | CTCAAACGCAGGTATTCAGC | TTGTAGAGGCCAGTTGCAGT |
